# Identification of the Trace Components in BopuzongJian and *Macleaya cordata* Extract Using LC-MS Combined with a Screening Method

**DOI:** 10.3390/molecules26133851

**Published:** 2021-06-24

**Authors:** Zhuang Dong, Mengting Liu, Xiaohong Zhong, Xiaoyong Ou, Xuan Yun, Mingcan Wang, Shurui Ren, Zhixing Qing, Jianguo Zeng

**Affiliations:** 1 Hunan Key Laboratory of Traditional Chinese Veterinary Medicine, Hunan Agricultural University, Changsha 410128, China; dzlebron0701@163.com (Z.D.); lmt19970808@163.com (M.L.); ouxiaoyong123456@163.com (X.O.); yunxuan0416@163.com (X.Y.); canming811@163.com (M.W.); renshurui0701@163.com (S.R.); 2 College of Horticulture, Hunan Agricultural University, Changsha 410128, China; xh-zhong@163.com; 3 College of Veterinary Medicine, Hunan Agricultural University, Changsha 410128, China

**Keywords:** trace component analysis, BopuzongJian, *Macleaya cordata* extract, isoquinoline alkaloids, screening method, LC-MS

## Abstract

BopuzongJian and *Macleaya cordata* extract were developed from *Macleaya* *cordata* and are widely used in agriculture and animal husbandry, but their trace components have been rarely reported in the literature. Trace component analysis is of great importance to the quality and safety of veterinary drugs. In this study, high-performance liquid chromatography/quadrupole time-of-flight mass spectrometry (HPLC-Q-TOF-MS) combined with a screening method was used to screen and characterize the trace components in BopuzongJian and *Macleaya cordata* extract. A total of 58 trace components were screened from BopuzongJian and *Macleaya cordata* extract using the screening strategies, of which 39 were identified by their accurate *m/z* value, characteristic MS/MS data, and fragmentation pathways of references. This established method was used for trace component analysis for the first time and proved to be a useful and rapid tool to screen and identify the trace components of BopuzongJian and *Macleaya cordata* extract, especially for those at trace levels in a complex sample. In addition, this study marks the first comprehensive research into trace components in these two products and has great significance for the systematic detection of trace components in other plant-derived drugs.

## 1. Introduction

BopuzongJianSan^®^ and BoluohuiSan^®^ are natural plant-derived drug feed additives made from isoquinoline alkaloids extracted from natural Macleaya cordata, and can reduce the use of antibiotics and chemical drugs in food animals. BoluohuiSan^®^ is the first Chinese veterinary medicine feed substitute, and has been used stably in more than 70 countries and regions for more than ten years. BopuzongJian and Macleaya cordata extract are raw materials of BopuzongJianSan^®^ and BoluohuiSan^®^, respectively, which are widely used in the animal breeding industry as a kind of safe, effective, and controllable Chinese veterinary medicine [1,2,3,4,5]. BopuzongJian is gray-white powder, and its main effect is anti-inflammatory, which means that it can be used to treat chicken *Escherichia coli* diarrhea [2]. The main active components are protopine and allocryptopine, and the total content is not less than 50% [6]. *Macleaya cordata* extract is an orange powder with a pungent odor, and its main effects are anti-inflammatory, thereby allowing for the maintenance of intestinal health [7]. The main active components of *Macleaya cordata* extract are sanguinarine and chelerythrine, with a total content of not less than 60% [6]. However, as of yet, there has been no systematic study on the trace components in these two products, which makes it difficult for us to correctly assess the influence of trace component composition.

The presence of trace components in veterinary drugs reduces their activity, affects their stability, and even produces adverse reactions [8]. Trace Components have an important effect on the quality and safety of drugs [9,10]. Therefore, the control of trace components is an important part of ensuring the quality of veterinary drugs. However, identifying the trace components from the complex matrix of plant-derived drugs remains a challenge for current analytical techniques. Many instrumental testing methods have been used in previous studies, including gas chromatography–mass spectrometry (GC-MS) [11], liquid chromatography–diode array detection (LC-DAD) [12,13], LC–mass spectrometry (LC-MS) [14,15], capillary electrophoresis–mass spectrometry, and LC–nuclear magnetic resonance (NMR) [15]. LC-MS is playing an increasingly important role in identifying trace components because of its high efficiency of separation, low level of sample consumption, excellent sensitivity, strong specificity, and the ability to provide a wealth of structural information [16,17]. However, it is time consuming to manually search for trace component signals from large amounts of raw MS data, and the signals of trace trace components are easily omitted, especially those submerged by background ions [18,19,20] These difficulties are considered as the bottleneck of screening trace components when using LC-MS. A robust component-mining method is needed to rapidly and efficiently screen trace components.

In many early studies [21,22], the first step of identifying compounds using LC-MS is to establish the total ion chromatograms (TICs) of the samples. Through the analysis of the MS and MS/MS data of each mass spectrum peak on the TIC, the structures can be preliminarily inferred and identified. However, compounds with obvious peaks in the TICs can easily be detected and identified, while it is difficult to identify and characterize components with trace amount [23]. To solve this problem, a screening strategy including the non-target, accurate-target, and extensive-target method combined with LC-MS was employed for systematical screening trace components in the presence of BopuzongJian and *Macleaya cordata* extract. The trace components were then further determined by their characteristic MS/MS data and the fragmentation pathways of isoquinoline alkaloids [24]. Finally, a total of 58 trace components were screened, and 39 of them were identified from both products (Table 1).

## 2. Result and Discussion

### 2.1. Establishment of the Screening Method

LC-MS applies to the analysis of complex matrix samples with high detection sensitivity, strong separation ability, and flexibility [25], and it has been widely used in many fields, such as medicine and food analysis. In specific plant-derived extracts, the levels of compounds are different; high-content substances or metabolites that display obvious peaks in the TIC can easily be detected, while it is usually difficult to identify low levels of compounds [26]. Therefore, the establishment of a comprehensive and systematic method for the detection of compounds from TICs is a crucial step before using LC/MS to identify these trace components.

**Figure 1:**
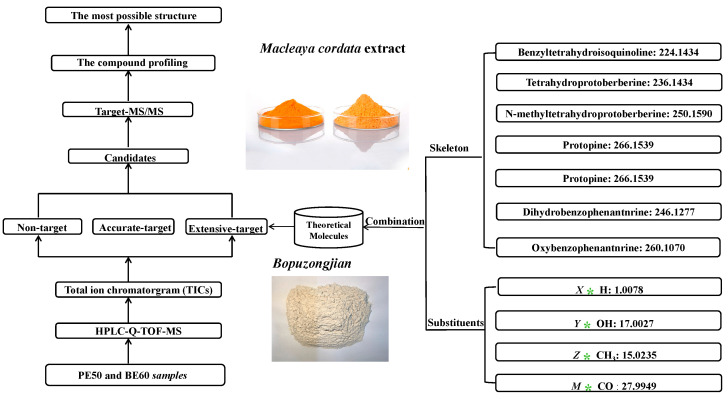
Diagram of systematic screening the trace components from BopuzongJian and *Macleaya cordata* extract. *X*, *Y*, *Z*, and *M* represent the number of hydrogen (H), hydroxy (OH), methyl (CH_3_) and carbon monoxide (CO), respectively.

**Figure 2:**
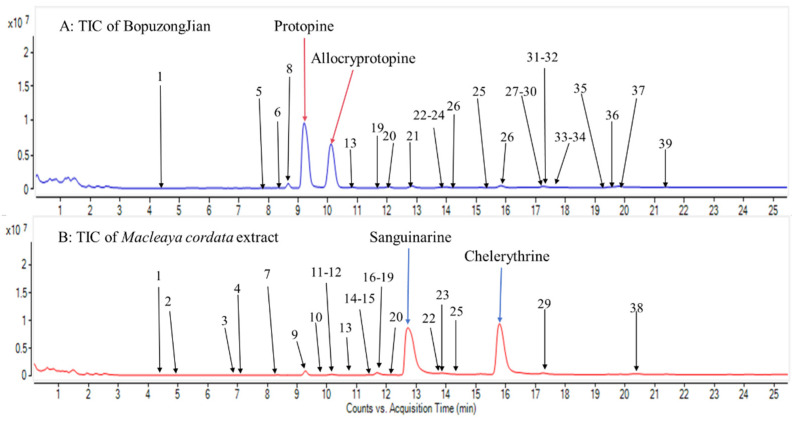
Total ion chromatograms (TICs) of (**A**) BopuzongJian and (**B**) *Macleaya cordata* extract.

**Figure 3:**
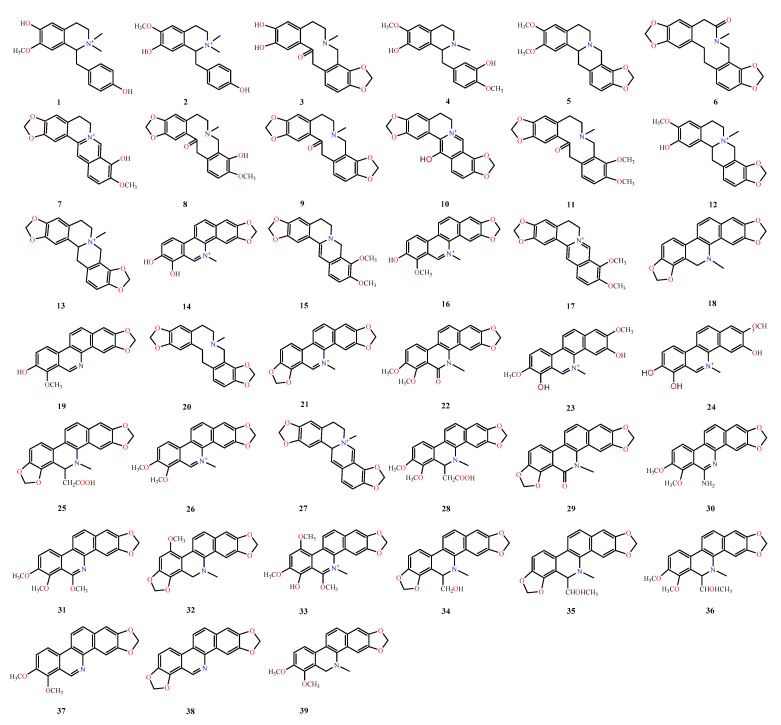
Structures of identified trace components from BopuzongJian and *Macleaya cordata* extract.

In this study, we proposed three strategies, namely, non-, accurate-, and extensive-target methods, to detect and identify trace components in BopuzongJian and *Macleaya cordata* extract (Figure 1). The non-target method is widely used as a traditional and common means to screen compounds one by one based on the distinct peaks in the TIC. However, some trace components that do not show significant peaks in the TIC are easily missed [27]. In this study, nine compounds were screened using the non-target method, and eight of them were identified. The accurate-target method is a means that is performed by first developing a list of compounds from the same genus as that in previous studies, including their accurate *m/z* value, molecular formula, and structure, to screen compounds that have been reported. The measured exact masses of candidates are acquired using the extracted-ion chromatogram (EIC) of the accurate *m/z* value of reported compounds in the TICs. However, this method is only applicable to well-known compounds; it is unsuitable for unknown compounds [27]. Through this method, 147 previously reported compounds were summarized (Table S1); 58 trace components were detected in both products using the accurate-target method; and 26 of them were identified. The extensive-target method is a relatively comprehensive method for screening possible analogues of well-known compounds in TICs. In a previous study [24], 19 types of isoquinoline alkaloids with different skeletons were reported in the genus *Macleaya*. These skeletons and the common substituent groups of the above 19 isoquinoline alkaloids were freely combined to form a total of 1084 theoretical *m/z* values. Then, the EICs of the formed theoretical exact masses on the TICs of the samples were determined. If the measured MS data matched the theoretical *m/z* values, those combined molecules were considered to exist in the sample. In addition, the mass spectrometry analysis became easier, because the skeleton and substituents of the theoretical molecular weight were explicit. Finally, 39 trace components were screened based on the extensive-target method (Figure 2), 26 of which were further identified. In this study, 58 trace components were screened from the BopuzongJian and *Macleaya cordata* extract by combining the above three methods, and 39 of them were identified on the basis of their characteristic MS/MS spectra (Figure 3).

### 2.2. Screening and Identification of Benzyltetrahydroisoquinoline-Type Alkaloids

**Figure 4:**
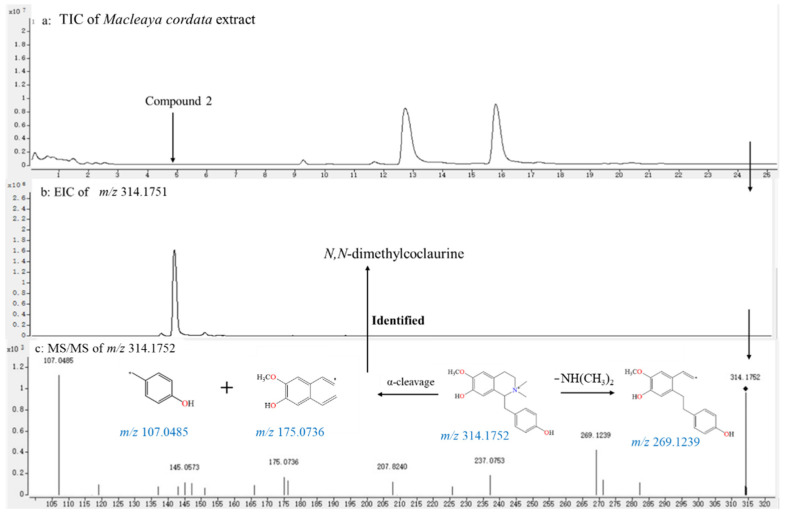
Screening of compound **2** by accurate-target method in the TIC of *Macleaya cordata* extract and determination of its structure by the characteristic MS/MS spectrum. (**a**) TIC of *Macleaya cordata* extract; (**b**) EIC of *m/z* 314.1751; (**c**) MS/MS spectra of *m/z* 314.1752.

The accurate-target method was the main tool for screening benzyltetrahydroisoquinoline-type alkaloids in BopuzongJian and *Macleaya cordata* extract, which were isolated and determined from the genus *Macleaya*, and their theoretical *m/z* values were preferentially employed to screen trace components in the TICs of both products. These candidates were further determined by their characteristic MS/MS data and the fragmentation pathways of benzyltetrahydroisoquinoline alkaloids. Taking compound **2** (a reported alkaloid from *M. cordata*) as an example, it was difficult to detect alkaloid **2** using the non-target method because of the lack of distinct peak in the TICs. An obvious peak was obtained by the EIC of theoretical exact mass (*m/z* 314.1751) in the TIC of *Macleaya cordata* extract. The MS/MS spectrum of alkaloid **2** was further obtained by the target MS/MS method and was in accordance with the fragmentation behaviors of benzyltetrahydroisoquinoline alkaloids [24]. We observed the loss of NH(CH_3_)_2_ moiety from the protonated ion at *m/z* 314.1751 and the formation of the high abundance fragment ion at *m/z* 269.1239, which indicated that two methyl groups were connected to a N atom. The fragments at *m/z* 175.0736 was formed by α-cleavage, which demonstrated a methoxyl and a hydroxyl were assigned to the A-ring. The ion at *m/z* 107.0485 was generated by β-cleavage, which indicated that a hydroxyl was connected to the C-ring. Therefore, alkaloid **2** was tentatively identified as *N,N*-dimethylcoclaurine by the above fragmentation behaviors (Figure 4).

Taking compound **4** (Rt = 7.114 min) as another example, it was also difficult to identify compound **4** using the non-target method due to its low abundance peak in the TIC. The EIC of theoretical mass (*m/z* 330.1700) of a reported compound in the TIC of *Macleaya cordata* extract was determined. Its measured MS data in positive mode were *m/z* 330.1440, which indicated that the reported compound may exist in *Macleaya cordata* extract, and the MS/MS spectrum was further obtained by the target MS/MS method. In the MS/MS spectrum of this trace component, the fragment ions at *m/z* 299.1172 was produced by the neutral loss of NH_2_CH_3_ moiety from the ion at *m/z* 330.1440, which demonstrated that a methyl groups was connected to a N atom. The fragment ions at *m/z* 137.0616 and 192.1009 were produced by α-cleavage from the ions at *m/z* 330.1440. These characteristic MS/MS data are consistent with the structure of the reported compound [27]. Therefore, compound **4** was tentatively identified as reticuline (Figure S1). Using a similar method, compound **1** was screened and identified as *N,N*-dimethylisococlaurine.

### 2.3. Screening and Identification of Protopine-Type Alkaloids

**Figure 5:**
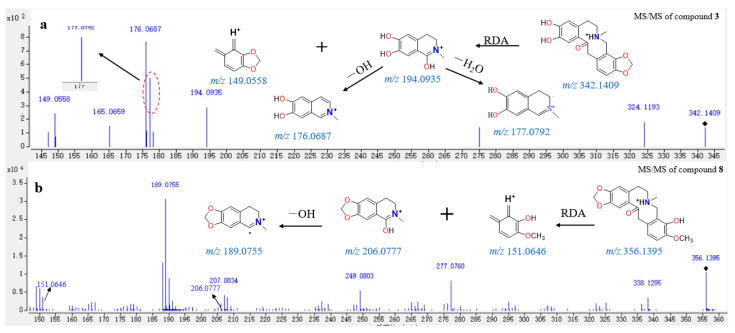
MS/MS spectra of compounds **3** (**a**) and **8** (**b**) and corresponding fragmentation behaviors.

The common substituent groups of protopine alkaloids are methylenedioxy (*m/z* value: 46.0055), methoxy (31.0184), hydroxyl (17.0027), and glucosyl (179.0556). There are four substitution sites on the skeleton; therefore, the four substituent groups of protopine alkaloids are connected to the four substitution sites in permuted and combined manner, forming a series of 88 theoretical accurate *m/z* values (Table S2). Then, the EIC of the theoretical *m/z* values on the TIC of BopuzongJian and *Macleaya cordata* extract were determined, and the candidates were further characterized by their MS/MS data. Taking compound **3** (Rt = 6.945 min) as an example, it was difficult to detect alkaloid **3** with non-target means due to the low-abundance peak in the TIC. However, this compound was identified by the extensive-target method using the EIC of theoretical *m/z* values (342.1409) on the TIC of BopuzongJian. In the extensive-target method, the structure of alkaloid **3** was formed by a protopine skeleton, one methylenedioxy group, and two hydroxyl groups. In the MS/MS spectrum of compound **3**, it can be seen that there is a fragment ion at *m/z* 194.0812, and this fragment ion continues to lose an OH radical or H_2_O to form highly abundant fragment ions at *m/z* 177.0792 or 176.0687. The above fragmentation pathways show that the A-ring of compound **3** contains two adjacent hydroxyl groups. The fragment ions at *m/z* 149.0558 and 165.0659 were formed in the MS/MS spectra of alkaloid **3**, indicating that the d-ring of compound **3** contains a methylenedioxy group. Therefore, compound **3** was identified as demethylcryprotopine [24] (Figure 5). Using the same method, compounds **11** and **20** were identified as allocryprotopine and isoprotopine, respectively.

Compound **8** (Rt = 8.677 min) was screened with the non-target and accurate-target methods simultaneously due to the obvious peak in the TIC of BopuzongJian, and it was isolated and determined from the genus *Macleaya*. The EIC of theoretical mass (*m/z* 356.1492) of the reported compound in the TIC of BopuzongJian was determined. In the MS/MS spectrum of compound **8**, the fragment ions at *m/z* 206.0777 and 151.0646 were produced by Retro–Diels–Alder reaction from the ions at *m/z* 356.1359. The fragment ions at *m/z* 188.0709 and 189.0755 was formed by the neutral loss of H_2_O moiety and the OH radical from the ion at *m/z* 206.0777, respectively. The MS/MS data of the screened compound **8** agree with the structural character of the reported alkaloid [28,29]. Therefore, compound **8** was tentatively identified as dimethyl-allocryprotopine (Figure 5). Compound **9** was identified as protopine by comparing the retention time, MS, and MS/MS data with the corresponding standards.

### 2.4. Screening and Identification of Tetrahydroproberberine-Type Alkaloids

The extensive-target method plays a principal role in the screening of tetrahydroproberberine-type alkaloids in BopuzongJian and *Macleaya cordata* extract. The substituent groups of tetrahydroproberberine-type alkaloids mainly include methylenedioxy (*m/z* value 46.0055), methoxy (31.0184), hydroxyl (17.0027), and glucosyl (179.0556). There are four substitution sites on the skeleton from the structural characteristics of tetrahydroproberberine-type alkaloids. Therefore, the four substituents of the tetrahydroproberberine-type alkaloid are connected to the four substitution sites on the skeleton in a permutated and combined manner, forming 88 theoretical accurate molecular weights. Taking compound **5** (Rt = 7.911 min) as an example, we set the theoretically accurate *m/z* value at 340.1543 in the TIC of BopuzongJian and *Macleaya cordata* extract, and its MS/MS spectra was obtained using the target MS/MS method. It can be observed from the MS/MS spectrum of compound **5** that there is a highly abundant molecular ion peak of *m/z* 192.1101, indicating that adjacent methoxy groups exist in the A-ring. The presence of low-abundance *m/z* 149.0652 fragment ions indicated that the d-ring contains a methylenedioxy. On the basis of the above fragmentation pathways [24,28], compound **5** was tentatively identified as isotetrahydroproberberine (Figure S1). Using the same method, compounds **12** and **13** were preliminarily identified as *N*-methylpyrophylline and *N*-methyltetrahydropalmatine, respectively.

It was difficult to characterize compound **6** (Rt = 8.31 min) using the non-target method because of the poor response in the TIC. However, this compound was easily detected by the accurate-target method. The EIC of theoretical exact mass (*m/z* 324.1230) of a reported compound in the TIC of BopuzongJian was determined, and its measured MS data in positive mode were at *m/z* 314.1214 and were subsequently obtained using the target-MS/MS method, which indicated that the reported compound may exist in BopuzongJian. In the MS/MS spectrum of compound **6**, the fragment ions occurring at *m/z* 176.0779 and 149.0579 were formed by the B-ring cleavage reaction from protonated ion at *m/z* 324.1214. Moreover, the fragment ion occurring at *m/z* 294.1161 was produced by the loss of CH_2_O from the ion at *m/z* 324.1214. The above fragmentation behaviors agree with the structural character in the previous reports of tetrahydroproberberine-type alkaloids [28,29]. Therefore, compound **6** was identified as tetrahydrocoptisine (Figure S1).

### 2.5. Screening and Identification of Protoberberine-Type Alkaloids

The accurate- and extensive-target methods were mainly used to detect protoberberine-type alkaloids in BopuzongJian and *Macleaya cordata* extract due to the low-abundance peak in the TICs. A total of 15 protoberberine-type alkaloids from the genus *Macleaya* (Table S1) and 1 candidate (compound **17**) were detected in the TICs of *Macleaya cordata* extract using the accurate-target method. This candidate was further determined by its characteristic MS/MS data. In the MS/MS spectrum of compound **17** (Rt = 11.699 min, *m/z* 336.1154 [M + H]^+^), the high-abundance fragment ions mainly appeared at the relatively high *m/z* values region, which indicated that the main fragmentation behavior was loss of substituent groups from the protonated ion, and the skeleton was difficult to cleavage. The above fragmentation pathway is in accordance with the structural character of protoberberine-type alkaloid [24,28,29]. In addition, fragment ions at *m/z* 320.0759, 292.0937, and 318.0738 were observed, and these were generated by the loss of CH_4_, CO, and 2H from the protonated ions at *m/z* 336.0858, 320.0759, and 320.0759, respectively. These characteristic fragments indicated that alkaloid **17** was berberine (Figure S2).

The extensive-target method was employed to detect other protoberberine-type alkaloids. The main substituents of this type of alkaloid in *Macleaya* plants are methylenedioxy (theoretical *m/z* value: 46.0055), methoxy (31.0184), hydroxyl (17.0027), and glucose (179.0556). On the basis of the structural characteristics of protoberberine alkaloids, four common substitution sites were present on the skeleton. Therefore, the four substituent groups were added to the four different substituent sites in a permutated and combined manner. A total of 88 theoretical accurate *m/z* values were produced (Table S2), and 3 possible protoberberine alkaloids (**7**, **10**, and **13**) were obtained through the EIC of these exact *m/z* values on the TICs of BopuzongJian and *Macleaya cordata* extract. Their structures were tentatively identified by characteristic MS/MS spectra and the well-investigated fragmentation pathway of protoberberine alkaloids. Taking compound **7** (Rt = 8.393 min, *m/z* 322.0909 [M + H]^+^) as an example, the different *m/z* values between alkaloids **7** and **17** was 13.9949 Da, which indicated that an OCH_3_ group was replaced by an OH group. In the MS/MS spectrum of compound **7**, the loss of a CH_3_ radical from the protonated parent ion at *m/z* 322.0909 and the formation of high-abundance fragments at *m/z* 307.0822 were noted; however, the neutral loss of CH_4_ moiety was not observed, which further indicated that the adjacent methoxyl was replaced by adjacent hydroxyl and methoxyl [24,28]. Therefore, compound **7** was tentatively identified as isothalonil (Figure S2). Using a similar method, the remaining protoberberine-type compounds **10** and **13** were screened and identified as 13-hydroxyl-coptisine and N-methyltetrahydropalmatine, respectively.

### 2.6. Screening and Identification of Benzophenanthrine-Type Alkaloids

Non-target, accurate-target, and extensive-target means were used to simultaneously screen benzophenanthrine-type alkaloids from the TICs of BopuzongJian and *Macleaya cordata* extract. The identification of these screened compounds by fragmentation pathways of references was performed. Taking compound **29** (Rt = 17.268 min, *m/z* 348.0962 [M + H]^+^**)** as an example, this alkaloid was mainly screened in the TICs of BopuzongJian and *Macleaya cordata* extract using the non-target method. The loss of the CH_3_ radical and CO moiety from the mother ion at *m/z* 348.0962 and the formation of the high-abundance fragment ions at *m/z* 333.0718 and 305.0666 were observed in the MS/MS spectrum of alkaloid **29**, which indicated that the CH_3_ and CO groups were included in the structure of compound **29**. The above fragmentation behaviors were consistent with the structural character of oxysanguinarine (Figure S3), which has previously been reported in the genus *Macleaya*. Using the same method, the benzophenanthrine-type alkaloids **16**, **18**, **21**, **26**, and **37**, which have higher abundance peaks, were tentatively screened and identified as demethylated chelerythrine, dihydrosanguinarine, sanguinarine, chelerythrine, and diazomethylchelerythrine, respectively (Figure 4).

Other benzophenanthrine-type alkaloids are difficult to screen and detect due to the fact that there are no obvious peaks in their TICs. Therefore, the accurate-target and extensive-target methods played an important role. The main substituents of benzophenanthridine alkaloids in *Macleaya* plants were methylenedioxy (*m/z* value, 46.0055), methoxy (31.0184), hydroxyl (17.0027), and glucose (179.0556). The four substituents of benzophenanthridine alkaloids were connected to the four substituent sites on the skeleton through permutation and combination, forming 154 theoretical accurate *m/z* values. In addition, these benzophenanthridine alkaloids were characterized on the basis of previous studies employing the accurate-target method (Table S1). A total of 19 possible benzophenanthridine alkaloids were found using the EIC of the 154 theoretical exact *m/z* values. Finally, 17 benzophenanthridine alkaloids were screened and identified by the target MS/MS data. Taking compound **39** (Rt = 21.384 min) as an example, the EIC of theoretical mass (*m/z* 350.1387) in the TIC of BopuzongJian was determined. In the MS/MS spectrum of compound **39**, fragment ions were produced mainly by the loss of some substituent groups. The high-abundance ion at *m/z* 335.1108 formed as a consequence of the loss of a CH_3_ fragment from the ion at *m/z* 350.1306. The ion at *m/z* 349.1318 was produced by the loss of a H atom from the precursor ion at *m/z* 350.1306. The proposed fragmentation pathways of compound **39** are shown in Figure S3, and they agree with the previous findings in the literature [28,29]. Therefore, this compound was tentatively identified as dihydrochelerythrine. Using the same method, other benzophenanthrine-type alkaloids, including compounds **14**, **19**, **22**, **23**, **24**, **25**, **27**, **28**, **30**, **31**, **32**, **33**, **34**, **35**, **36**, and **38,** were screened and identified (Table 1, Figure S4).

A total of 58 trace components were detected from BopuzongJian and *Macleaya cordata* extract using the above screen strategies; 39 of them were identified by their accurate *m/z* value and characteristic MS/MS data. The pharmacological activities, degradation, and residue of the four main alkaloids (including protopine (**9**), allocryptopine (**11**), sanguinarine (**21**), and chelerythrine (**26**)) have been well investigated [30,31]; however, the remaining alkaloids require further study. To further assess the bioactivities and adverse reaction of these trace components in animals, it is necessary to obtain sufficient amounts through synthesis methods. The synthetic routes of tetrahydroprotoberberine, protoberberine, protopine, and benzophenanthridine-type alkaloids, which were screened and identified from both products, were well developed [32].

## 3. Experimental

### 3.1. Materials and Reagents

Acetonitrile and formic acid (HPLC-grade) were purchased from Merck (Darmstadt, Germany) and ROE (Newark, DE, USA), respectively. Methanol (AR) was purchased from the National Institutes for Food and Drug Control (Beijing, China). Deionized water was purified using a Milli-Q system (Bedford, MA, USA). All solvents used for HPLC-Q-TOF-MS and HPLC-Q-TOF-MS/MS analysis were of the highest available analytical grade.

### 3.2. HPLC Conditions

Chromatography was performed using an Aglient 1290 HPLC system (Aglient Technologies, Palo Alto, CA, USA). The separation was performed on an XAqua C_18_ column (150 mm × 2.1 mm, 5 μm; Accrom Technologies Co. Ltd., China). Then, 0.1% formic acid aqueous solution (*v*/*v*, solvent A) and 0.1% FA/ACN (*v*/*v*, solvent B) were used as an elution system, and the gradient elution program was optimized as follows: 0–1 min, 5–10% B; 1–6 min, 10–30% B; 6–7 min, 30–38% B; 8–15 min, 38–60% B; 16–25 min, 75–95%. The column temperature was maintained at 35 °C, and the injection volume was 1 μL. The flow rate was set at 0.3 mL/min, and the detection wavelength was 280 nm.

### 3.3. Q-TOF-MS Conditions

Mass spectrometric experiments were performed using 6530 quadrupole time-of-flight mass spectrometry (Q-TOF-MS). The TOF data were acquired in positive ionization (ESI^+^), and the mass range was between *m/z* 100 and 1000. The conditions of the Q-TOF-MS were optimized as follows: gas temperature, 345 °C; drying gas, 10 L/min; nebulizer gas (N_2_) pressure, 45 psi; sheath gas (N_2_) temperature, 350 °C; sheath gas flow rate, 11 L/min; vcap voltage, 4000 V; skimmer voltage, 65 V; fragmentor voltage, 175 V; OCT1 RF Vpp, 750 V. Purine (C_5_H_4_N_4_, [M+H]^+^ ion at *m/z* 121.0508) and HP-0921 (C_18_H_18_O_6_N_3_P_3_F_24_, [M + H]^+^ ion at *m/z* 922.0097) were used to obtain high-accuracy mass calibration, and automated calibration was used to ensure mass correction during sample analysis. The targeted MS/MS experiments were operated using variable collision energy (20–45 eV), which was optimized for each compound. All data (MS and MS^2^) were processed using the data explorer software of the Q/TOF instrument.

### 3.4. Sample Preparation

The samples of BopuzongJian and *Macleaya cordata* extract were obtained from Micolta Bioresource Inc., Ltd. The *Macleaya cordata* plants used were from Xinning County, Hunan Province, China. The preparation process of these two raw materials was as follows: *Macleaya cordata* was extracted via percolation with acidic solution, and the extract was adjusted to an alkaline solution for precipitation. After being dissolved in ethanol, reflux extraction was carried out. The obtained ethanol extract was added to acid for precipitation. After the residual acid was washed with ethanol and dried, the *Macleaya cordata* extract was obtained.

The filtrate, after alcohol extraction and acid precipitation, was concentrated under vacuum until there was no alcohol flavor. Then, it was heated to 60 °C for 1 h after the addition of water and filtration. The filtrate was subjected to alkali precipitation, and the precipitation was obtained. The filtrate was precipitated by the alkali; then, the BopuzongJian was obtained after vacuum drying and crushing.

Approximately 20 mg powder of both products was placed in a 50 mL volumetric flask, fully dissolve with 80% ethanol, and diluted to scale. After the solution had been centrifuged at 12,000 rpm for 15 min, the supernatant was filtrated through a 0.22 µm filter membrane and then injected into the HPLC-Q-TOF-MS system for analysis.

## 4. Conclusions

In this work, LC-MS combined with a systematic screening method, which includes non-, accurate-, and extensive-target approaches, was employed as a rapid and efficient analytical tool for the detection and identification of trace components in BopuzongJian and *Macleaya cordata* extract. Using this method, the existence of trace components could be efficiently determined even when signals for trace components were obscured by background ions or drug ions. A total of 58 trace components were screened from the 2 products, of which 39, including 3 benzyltetrahydroisoquinolines, 5 protopines, 4 tetrahydroptotoberberines, 4 protoberberines, and 23 benzophenanthridines, were identified by accurate *m/z* values, characteristic MS/MS data, and fragmentation pathways of references. The quantities of benzophenanthridine-type and protopine-type alkaloids were the largest, which is consistent with the distribution of these two types of alkaloids in the genus *Macleaya*. The study provides reference value for the quality evaluation of these two products and marks the first comprehensive study of trace components in BopuzongJian and *Macleaya cordata* extract. This method is rarely applied to the analysis of trace components in plant-derived drugs; therefore, the established screening strategies are of great significance to other plant-derived drugs to systematically detect bioactive metabolites in complex biological substrates.

## Figures and Tables

**Table 1:** Peak number (PN), retention time (T_R_), molecular formula, MS^1^, characteristic MS/MS ions, tentative identification, screening method, type, and source of trace components. Those compounds were screened by the non-target method (X), accurate-target method (Y), and extensive-target method (Z).

PN	T_R_ (min)	Molecular Formula	MS^1^	Characteristic MS/MS Ions (*m/z*)	Tentative Identification	Screening Method	Type	Source
1	4.391	C_19_H_24_NO_3_^+^	314.1744	269.1149, 237.0797, 175.0934, 143.0482, 107.0481	*N, N*-dimethylisococlaurine	Y	Benzyltetrahydroisoquinoline	BopuzongJian/*Macleaya cordata* extract
2	4.915	C_19_H_24_NO_3_^+^	314.1752	269.1239, 207.0753, 175.0736, 107.0485	*N, N*-dimethylcoclaurine	Y	Benzyltetrahydroisoquinoline	*Macleaya cordata* extract
3	6.945	C_19_H_20_NO_5_^+^	342.1409	324.1193, 194.0935, 177.0792, 176.0687, 165.0659	Demethylcryptopine	Z	Protopine	*Macleaya cordata* extract
4	7.114	C_19_H_24_NO_4_^+^	330.144	299.1172, 192.1009, 137.0616	Reticuline	Y	Benzyltetrahydroisoquinoline	*Macleaya cordata* extract
5	7.911	C_20_H_22_NO_4_^+^	340.1258	192.1101, 149.0652	Isotetrahydroprotoberberine	Z	Tetrahydroptotoberberine	BopuzongJian
6	8.310	C_19_H_18_NO^4+^	324.1214	176.0779, 149.0579, 294.1161	Tetrahydrocoptisine	Y	Tetrahydroptotoberberine	BopuzongJian
7	8.393	C_19_H_16_NO_4_^+^	322.0909	279.0869, 294.1065, 307.0822,	Isothalonil	Z	Protoberberine	*Macleaya cordata* extract
8	8.677	C_20_H_22_NO_5_^+^	356.1395	151.0646,188.0709,189.0755,206.0777	Demethyl allocryprotopine	XY	Protopine	BopuzongJian
9	9.201	C_20_H_20_NO_5_^+^	354.1351	336.1232, 189.0769, 206.0803,149.0577	Protopine	XY	Protopine	*Macleaya cordata* extract
10	9.891	C_19_H_14_NO_5_^+^	336.0862	318.0726, 308.0850, 290.0797	13-hydroxyl-coptisine	Z	Protoberberine	*Macleaya cordata* extract
11	10.116	C_21_H_24_NO_5_^+^	370.1584	352.1580, 206.0812, 188.0728, 189.0893	Allocryprotopine	YZ	Protopine	*Macleaya cordata* extract
12	10.334	C_20_H_22_NO_4_^+^	340.1614	192.0924, 170.9604	*N*-methylpyrophylline	Z	Tetrahydroptotoberberine	*Macleaya cordata* extract
13	10.824	C_20_H_20_NO_4_^+^	338.1380	190.0845, 149.0669	*N*-methyltetrahydropalmatine	Z	Tetrahydroptotoberberine	BopuzongJian/*Macleaya cordata* extract
14	11.373	C_19_H_14_NO_4_^+^	320.0916	305.0672, 292.0863, 262.0825, 246.0860	Didemethyl chelerythrine	YZ	Benzophenanthridine	*Macleaya cordata* extract
15	11.414	C_20_H_20_NO_4_^+^	338.1378	323.1047, 322.1029, 294.0938	7,8-dihydroberberine	Z	Protoberberine	*Macleaya cordata* extract
16	11.639	C_20_H_16_NO_4_^+^	334.0927	319.0828, 304.0570, 291.0834, 276.0496	Demethylated chelerythrine	X	Benzophenanthridine	*Macleaya cordata* extract
17	11.699	C_20_H_18_NO_4_^+^	336.1154	320.0759, 318.0738, 292.0937	Berberine	Y	Protoberberine	*Macleaya cordata* extract
18	11.705	C_20_H_16_NO_4_^+^	334.1021	319.0836, 318.0738, 304.0545, 290.0758, 276.0665	Dihydrosanguinarine	X	Benzophenanthridine	*Macleaya cordata* extract
19	11.722	C_19_H_14_NO_4_^+^	320.0839	305.0701, 292.0909, 277.0703, 262.0830	Isodimethylchelerythrine	YZ	Benzophenanthridine	BopuzongJian/*Macleaya cordata* extract
20	12.039	C_20_H_20_NO_5_^+^	354.1297	206.0809, 189.0758, 188.0691, 149.0591	Isoprotopine	YZ	Protopine	BopuzongJian/*Macleaya cordata* extract
21	12.754	C_20_H_14_NO_4_^+^	332.0791	317.0701, 304.0924, 274.0836	Sanguinarine	X	Benzophenanthridine	BopuzongJian
22	13.911	C_21_H_18_NO_5_^+^	364.1168	349.0923, 348.0824, 334.0633, 320.0859, 306.0723	Oxychelerythrine	YZ	Benzophenanthridine	*Macleaya cordata* extract
23	13.928	C_20_H_18_NO_4_^+^	336.2800	321.0874, 320.0857, 306.0772, 304.0898, 292.0975	Dedimethyl-benzophenanthridinium	YZ	Benzophenanthridine	BopuzongJian/*Macleaya cordata* extract
24	13.944	C_20_H_18_NO_4_^+^	322.1045	321.0990, 306.1000, 278.1161	Detrimethyl-benzophenanthridinium	YZ	Benzophenanthridine	BopuzongJian
25	15.451	C_22_H_18_NO_6_^+^	392.1193	332.0920, 318.0768, 274.0667	6-acetoxy-dihydrosanguinarine	YZ	Benzophenanthridine	BopuzongJian
26	15.825	C_21_H_18_NO_4_^+^	348.2089	332.0823, 318.0620, 304.0910, 290.0654	Chelerythrine	X	Benzophenanthridine	BopuzongJian
27	17.135	C_23_H_22_NO_6_^+^	408.1354	348.1193, 333.0833	6-acetoxy-dihydrochelervthrine	YZ	Benzophenanthridine	BopuzongJian
28	17.248	C_29_H_24_NO_6_^+^	482.1590	467.1464, 452.1271, 422.0905, 163.0776	Maclekarpine E	YZ	Benzophenanthridine	BopuzongJian
29	17.268	C_20_H_14_NO_5_^+^	348.0962	333.0718, 305.0666	Oxysanguinarine	X	Benzophenanthridine	BopuzongJian/*Macleaya cordata* extract
30	17.290	C_20_H_17_N_2_O_4_^+^	349.1243	334.1036,333.0974,319.0810, 305.0997, 291.0863	6-amino-chelerythrine	YZ	Benzophenanthridine	BopuzongJian
31	17.340	C_21_H_18_NO_5_^+^	364.1158	349.0838,334.0721,320.0849, 306.0833	6-methoxy-diazomethylchelerythrine	YZ	Benzophenanthridine	BopuzongJian
32	17.435	C_21_H_18_NO_5_^+^	364.1152	349.0906, 348.0738, 334.0626, 332.4152, 319.0601	10-methoxy-dihydrosanguinarine	YZ	Benzophenanthridine	BopuzongJian
33	17.493	C_21_H_18_NO_5_^+^	364.1148	349.0883, 334.0663, 306.0689	10-methoxy-demethyl chelerythrine	YZ	Benzophenanthridine	BopuzongJian
34	17.505	C_21_H_18_NO_5_^+^	364.0917	349.0955, 334.0682	6-methylol-dihydrosanguinarine	YZ	Benzophenanthridine	BopuzongJian
35	19.366	C_22_H_20_NO_5_^+^	378.1420	360.1198, 345.0956, 330.1045, 318.0653	6-ethoxy-dihydrosanguinarine	YZ	Benzophenanthridine	BopuzongJian
36	19.628	C_23_H_24_NO_5_^+^	394.2107	376.1556, 361.1176, 345.1085, 334.2396	6-hydroxyethylchelerythrine	YZ	Benzophenanthridine	BopuzongJian
37	19.836	C_20_H_16_NO_4_^+^	334.0885	319.0801, 318.0736, 304.0592, 290.0742	Diazomethylchelerythrine	X	Benzophenanthridine	BopuzongJian
38	20.402	C_19_H_12_NO_4_^+^	318.0738	290.0801, 288.0630, 260.0672, 232.0742	Diazomethylsanguinarine	YZ	Benzophenanthridine	BopuzongJian
39	21.384	C_21_H_20_NO_4_^+^	350.1306	335.1108, 349.1318	Dihydrochelerythrine	YZ	Benzophenanthridine	BopuzongJian

## Supplementary Materials

The following are available online, Figure S1: MS/MS spectra of compound **4**, **5**, **6** and corresponding fragmentation behaviors. Figure S2: MS/MS spectra of compound **17** and **7** and corresponding fragmentation behaviors. Figure S3: MS/MS spectra of compound **29** and **39** and corresponding fragmentation behaviors. Figure S4: MS/MS spectra of the rest compounds; Table S1: The summarized alkaloids in genus *Macleaya* (*Macleaya cordata* and *Macleaya microcarpa).* Table S2: Protoberberine-type alkaloids skeleton, exact theoretical masses ([M + H]^+^), substituent groups (88 theoretical accurate *m/z* values).

## Sample Availability

Samples of the compounds are available from the authors.
