# Correction: Dong et al. Identification of the Trace Components in BopuzongJian and *Macleaya cordata* Extract Using LC-MS Combined with a Screening Method. *Molecules* 2021, *26*, 3851

**DOI:** 10.3390/molecules29061268

**Published:** 2024-03-13

**Authors:** Zhuang Dong, Mengting Liu, Xiaohong Zhong, Xiaoyong Ou, Xuan Yun, Mingcan Wang, Shurui Ren, Zhixing Qing, Jianguo Zeng

**Affiliations:** 1 Hunan Key Laboratory of Traditional Chinese Veterinary Medicine, Hunan Agricultural University, Changsha 410128, China; dzlebron0701@163.com (Z.D.); lmt19970808@163.com (M.L.); ouxiaoyong123456@163.com (X.O.); yunxuan0416@163.com (X.Y.); canming811@163.com (M.W.); renshurui0701@163.com (S.R.); 2 College of Horticulture, Hunan Agricultural University, Changsha 410128, China; xh-zhong@163.com; 3 College of Veterinary Medicine, Hunan Agricultural University, Changsha 410128, China

The article in question [1] reports the trace components of two products which are widely used in the animal breeding industry in China. However, some of the reported information could be misinterpreted; as such, we would like to clarify a few issues in order to avoid misleading the reader. We believe that it is important to state that the two products’ names and one of the pictures were incorrectly used.

Therefore, the following modifications should be considered: The terms “Bopu powder^®^ and Sangrovit^®”^ that appear in this paper should be replaced by “BopuzongJian” and “*Macleaya cordata* extract”. In addition, the word “Impurity” does not apply to plant-derived drugs, and it in this paper to describe the trace components should be replaced by “trace components”. In order to make this easier for the reader to follow, a short excerpt from the original manuscript is cited together with the respective modifications.

## 1. Incorrect Title

Original: 

Identification of the Impurities in Bopu Powder^®^ and Sangrovit^®^ by LC-MS Combined with a Screening Method.

This should be replaced with:

Identification of the trace components in BopuzongJian and *Macleaya cordata* extract using LC-MS Combined with a screening method.

## 2. Introduction

Original:

Bopu powder^®^ and Sangrovit^®^, whose main chemical compositions are isoquinoline alkaloids, are extracted from the natural plant *Macleaya cordata*, and they are widely used in the animal breeding industry as a kind of safe, effective, and controllable Chinese veterinary medicine [1–5].

This should be replaced with:

BopuzongJianSan^®^ and BoluohuiSan^®^ are natural plant-derived drug feed additives made from isoquinoline alkaloids extracted from natural *Macleaya cordata*, and can reduce the use of antibiotics and chemical drugs in food animals. BoluohuiSan^®^ is the first Chinese veterinary medicine feed substitute, and has been used stably in more than 70 countries and regions for more than ten years. BopuzongJian and *Macleaya cordata* extract are raw materials of BopuzongJianSan^®^ and BoluohuiSan^®^, respectively, which are widely used in the animal breeding industry as a kind of safe, effective, and controllable Chinese veterinary medicine [1–5].

## 3. Error in Figure

The names of the products “Bopu powder^®^” and “Sangrovit^®^” were incorrectly used. In this section, all mentions of “Bopu powder^®^” and “Sangrovit^®^” that appear in the three figures have been replaced by “BopuzongJian” and “*Macleaya cordata* extract”. In addition, the picture of “*Macleaya cordata* extract” in Figure 1 was incorrectly used, so we have made the appropriate changes.

These changes have no material impact on the aim of our paper. We apologize for any inconvenience caused to the readers and state that the scientific conclusions are unaffected. 

Figure 1

Original:

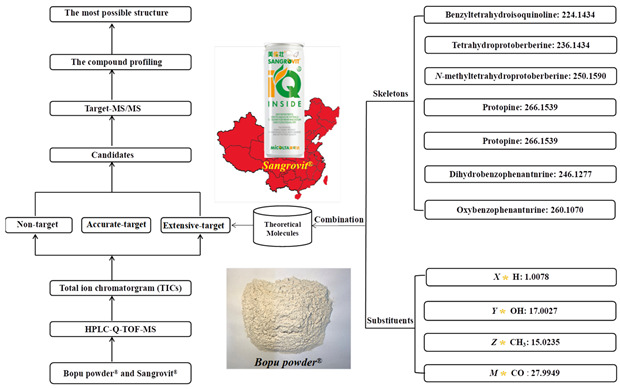



This should be replaced with:

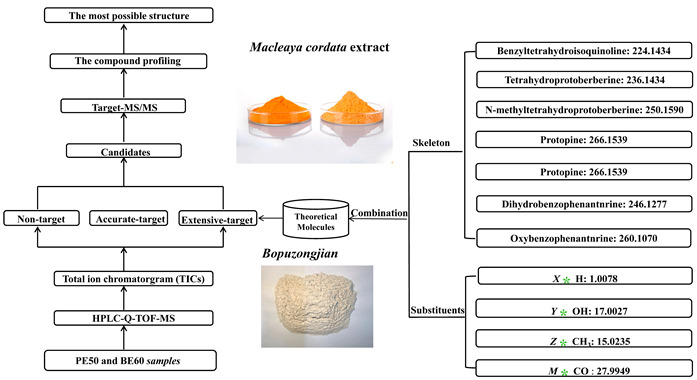



Figure 2

Original:

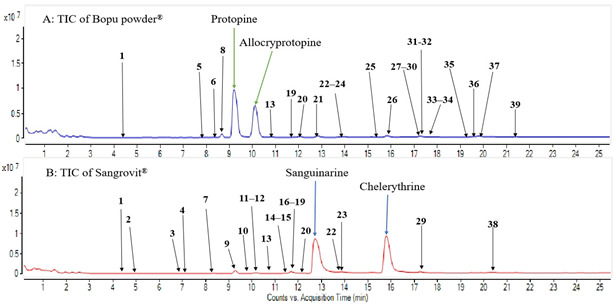



This should be replaced with:

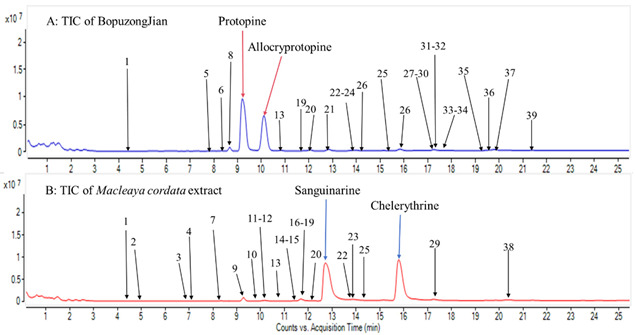



Figure 4

Original:

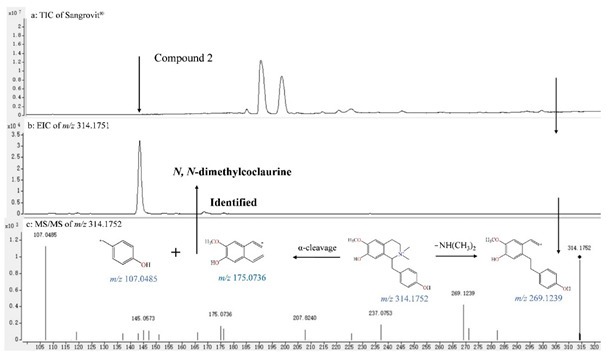



This should be replaced with:

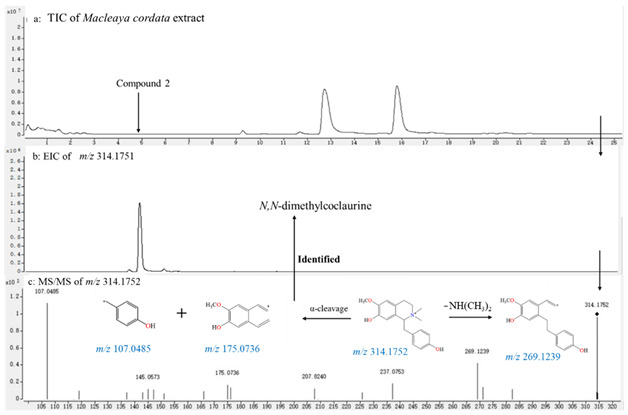



## 4. Sample Preparation

There was an error in the original article. The “Hunan Micolta Bioresource Co., Ltd.” was incorrectly written. Additionally, we have added more detailed product information in this section.

Original:

The samples of Bopu powder^®^ and Sangrovit^®^ were obtained from Hunan Micolta Bioresource Co., Ltd. and confirmed by Prof Jianguo Zeng (Hunan Agricultural University, China).

This should be replaced with:

The samples of BopuzongJian and *Macleaya cordata* extract were obtained from Micolta Bioresource Inc., Ltd. The *Macleaya cordata* plants used were from Xinning County, Hunan Province, China. The preparation process of these two raw materials was as follows: *Macleaya cordata* was extracted via percolation with acidic solution, and the extract was adjusted to an alkaline solution for precipitation. After being dissolved in ethanol, reflux extraction was carried out. The obtained ethanol extract was added to acid for precipitation. After the residual acid was washed with ethanol and dried, the *Macleaya cordata* extract was obtained.

The filtrate, after alcohol extraction and acid precipitation, was concentrated under vacuum until there was no alcohol flavor. Then, it was heated to 60 °C for 1 h after the addition of water and filtration. The filtrate was subjected to alkali precipitation, and the precipitation was obtained. The filtrate was precipitated by the alkali; then, the BopuzongJian was obtained after vacuum drying and crushing.

These changes have no material impact on the aim of our paper. We apologize again for any inconvenience caused to the readers and state that the scientific conclusions are unaffected. 
