# Supplementary material for: Identification of the Trace Components in BopuzongJian and Macleaya cordata Extract Using LC-MS Combined with a Screening Method
Source: Molecules. 2021 Jun 24;26(13):3851. doi: 10.3390/molecules26133851 (PMC8270340; doi:10.3390/molecules26133851)

Table S1. The summarized alkaloids in genus *Macleaya* (*Macleaya cordata* and *Macleaya microcarpa*).

No.	Alkaloids	Formula	Sources
1	α-allocryptopine/(±)-homechelidonine	$C_{21}H_{23}NO_5$	*M. cordata*<br>*M. microcarpa*
2	protopine	$C_{20}H_{19}NO_5$	*M. cordata*<br>*M. microcarpa*
3	β-allocryptopine	$C_{21}H_{23}NO_5$	*M. cordata*<br>*M. microcarpa*
4	cryptopine	$C_{21}H_{23}NO_5$	*M. cordata*<br>*M. microcarpa*
5	vaillatine	$C_{20}H_{23}NO_5$	*M. microcarpa*
6	2-demethylmuramine	$C_{21}H_{25}NO_5$	*M. microcarpa*
7	hunnemannine	$C_{20}H_{21}NO_5$	*M. microcarpa*
8	corycavidine	$C_{22}H_{25}NO_5$	*M. microcarpa*
9	muramine	$C_{22}H_{27}NO_5$	*M. microcarpa*
10	corycavamine	$C_{21}H_{21}NO_5$	*M. microcarpa*
11	2,3-didemethylcrytopine	$C_{19}H_{19}NO_5$	*M. cordata*
12	9,10-didemethyl-allcryptopine	$C_{19}H_{19}NO_5$	*M. cordata*
13	papaverine	$C_{20}H_{21}NO_4$	*M. cordata*
14	laudanine	$C_{20}H_{25}NO_4$	*M. cordata*
15	codamine	$C_{20}H_{25}NO_4$	*M. cordata*
16	pseudocodamine	$C_{20}H_{25}NO_4$	*M. cordata*
17	magnocurarine	$C_{19}H_{24}NO_3^+$	*M. cordata*
18	reticuline	$C_{19}H_{23}NO_4$	*M. cordata*
19	*N*-methylcoclaurine	$C_{18}H_{21}NO_3$	*M. cordata*
20	2-methoxylmagnocurarine	$C_{20}H_{26}NO_3^+$	*M. cordata*
21	colletine	$C_{20}H_{26}NO_3^+$	*M. cordata*
22	*N*-demethylcolletine	$C_{19}H_{23}NO_3$	*M. cordata*
23	sanguinarine	$C_{20}H_{14}NO_4^+$	*M. cordata*<br>*M. microcarpa*
24	chelerythrine	$C_{21}H_{18}NO_4^+$	*M. cordata*<br>*M. microcarpa*
25	chelirubine/bocconine	$C_{21}H_{16}NO_5^+$	*M. cordata*<br>*M. microcarpa*
26	macarpine	$C_{22}H_{18}NO_6^+$	*M. cordata*<br>*M. microcarpa*
27	chelilutine	$C_{22}H_{20}NO_5^+$	*M. cordata*<br>*M. microcarpa*
28	8-*O*-demethylchelerythrine	$C_{20}H_{16}NO_4^+$	*M. cordata*<br>*M.microcarpa*
29	benzophenanthridinium	$C_{22}H_{22}NO_4^+$	*M.microcarpa*
30	7,8-didemethylchelerythrine	$C_{19}H_{14}NO_4^+$	*M. cordata*
31	7-*O*-demethylchelerythrine	$C_{20}H_{16}NO_4^+$	*M. cordata*
32	norsanguinarine	$C_{19}H_{11}NO_4$	*M. cordata*<br>*M. microcarpa*
33	pancorine	$C_{20}H_{13}NO_5$	*M. microcarpa*
34	norchelerythrine	$C_{20}H_{15}NO_4$	*M. microcarpa*
35	6-methoxynorsanguinarine	$C_{20}H_{14}NO_5^+$	*M. cordata*
36	*O*-methylzanthoxyline	$C_{19}H_{15}NO_4$	*M. cordata*
37	10-methoxylnorchelerythrine	$C_{21}H_{18}NO_5^+$	*M. cordata*
38	oxychelerythrine	$C_{21}H_{17}NO_5$	*M. cordata*<br>*M. microcarpa*
39	oxysanguinarine/hydroxysanguinarine	$C_{20}H_{13}NO_5$	*M. cordata*<br>*M. microcarpa*
40	dihydroboccnine/dihydrochelirubine	$C_{21}H_{17}NO_5$	*M. cordata*

	/dihydrochelilutine		
41	oxybocconine/oxychelirubine	$C_{21}H_{15}NO_6$	*M. cordata*
42	bocconoline	$C_{22}H_{21}NO_5$	*M. cordata*
43	dihydrosanguinarine	$C_{20}H_{15}NO_4$	*M. cordata*
			M. microcarpa
44	ethoxysanguinarine	$C_{22}H_{19}NO_5$	*M. cordata*
			M. microcarpa
45	6-acetonyldihydrosanguinarine	$C_{23}H_{19}NO_5$	*M. cordata*
			M. microcarpa
46	6-methoxydihydrochelerythrine	$C_{22}H_{21}NO_5$	*M. cordata*
			M. microcarpa
47	dihydrochelerythrine	$C_{21}H_{19}NO_4$	*M. cordata*
			M. microcarpa
48	7-carbonyl-8-methoxy-5-methyl-2,3-(methylenedioxy)-benzo[c]phenanthridine	$C_{20}H_{16}NO_4$	*M. cordata*
49	6-butoxydihydrochelerythrine	$C_{24}H_{27}NO_5$	*M. microcarpa*
50	(5'R)-3'-methyl-2'(5'H)furanone-(5'→6)-(6S)-dihydrochelerythrine	$C_{26}H_{23}NO_6$	*M. microcarpa*
51	(2',6'-epoxy-1',2α,3′β,4α,5α-pentahydroxy)hexane-(1'→6)-dihydrochelerythrine	$C_{27}H_{29}NO_{10}$	*M. microcarpa*
52	6-(1'-hydroxyethyl)dihydrocheleythrine	$C_{25}H_{27}NO_5$	*M. microcarpa*
53	Spallidamine/6-carboxymethyl-dihydrosanguinarine	$C_{22}H_{17}NO_6$	*M. microcarpa*
			M. cordata
54	(5'R)-3'-methyl-2'(5'H)furanone-(5'→6)-(6S)-dihydrosanguinarine	$C_{25}H_{19}NO_6$	*M. microcarpa*
55	(5'R)-3'-methyl-2'(5'H)furanone-(5'→6)-(6R)-dihydrosanguinarine	$C_{25}H_{19}NO_6$	*M. microcarpa*
56	(1'E)-6-(3''-methoxy-4''-hydroxyphenyl)ethenyldihydrosan-guinarine/maclekarpine E	$C_{29}H_{23}NO_6$	*M. microcarpa*
			M. cordata
57	bis[6-(5,6-dihydrochelerythrinyl)]ether	$C_{42}H_{36}N_2O_9$	*M. microcarpa*
			M. cordata
58	6-methoxydihydrosanguinarine	$C_{21}H_{17}NO_5$	*M. cordata*
			M. microcarpa
59	6-acetonyldihydrochelerythrine	$C_{24}H_{23}NO_5$	*M. cordata*
60	sanguidimerine	$C_{43}H_{32}N_2O_9$	*M. cordata*
61	chelidimerine	$C_{43}H_{32}N_2O_9$	*M. cordata*
62	(±)-bocconarborine A	$C_{44}H_{36}N_2O_9$	*M. cordata*
			M. microcarpa
63	(±)-bocconarborine B	$C_{44}H_{36}N_2O_9$	*M. cordata*
64	angoline	$C_{22}H_{21}NO_5$	*M. cordata*
65	6α-iso-butanonyldihydrosanguinarine	$C_{24}H_{21}NO_5$	*M. cordata*
66	6α-iso-butanonyldihydrochelerythrine	$C_{25}H_{25}NO_5$	*M. cordata*
67	8-methoxydihydrosanguinarine	$C_{21}H_{17}NO_5$	*M. cordata*
68	8-hydroxydihychelerythrine	$C_{21}H_{19}NO_5$	*M. cordata*
69	(S)-6-(R-1-hydroxyethyl)-dihydrochelerythrine	$C_{23}H_{23}NO_5$	*M. cordata*
70	(R)-6-((R)-1-hydroxyethyl)-dihydrosanguinarine	$C_{22}H_{19}NO_5$	*M. cordata*
71	6α-(1-carboxymethyl)dihydrochelilutine	$C_{24}H_{23}NO_7$	*M. cordata*
72	(R)-6-((R)-1-hydroxyethyl)-dihydrochelerythrine	$C_{23}H_{23}NO_5$	*M. cordata*
73	6-carboxymethyldihydrochelerythrine	$C_{23}H_{21}NO_6$	*M. cordata*
74	8-methoxydihydrochelerythrine	$C_{22}H_{21}NO_5$	*M. cordata*
75	8-acetonyldihydrosanguinarine	$C_{23}H_{19}NO_5$	*M. cordata*
			M. microcarpa
76	8-acetonyldihydrochelerythrine	$C_{24}H_{23}NO_5$	*M. cordata*

			M. microcarpa
77	8-butanonyldihydrosanguinarine	$C_{24}H_{21}NO_5$	*M. cordata*
78	8-butanonyldihydrochelerythrine	$C_{25}H_{25}NO_5$	*M. cordata*
79	6-ethoxychelerythrine	$C_{23}H_{23}NO_5$	*M. cordata*
80	cordatine	$C_{22}H_{21}NO_5$	*M. cordata*
81	6-cyanodihydrosanguinarine	$C_{21}H_{14}N_2O_4$	*M. cordata*
82	6-methylol-7,8-demethyldihydro-chelerythrine	$C_{20}H_{17}NO_5$	*M. cordata*
83	6-hydroxyl-10-methoxylsanguinarine	$C_{21}H_{18}NO_6^+$	*M. microcarpa*
84	6-propoxysanguinarine	$C_{22}H_{20}NO_5^+$	*M. microcarpa*
85	6-propoxychelerythrine	$C_{23}H_{24}NO_5^+$	*M. microcarpa*
86	6-hydroxylchelerythrine	$C_{21}H_{20}NO_5^+$	*M. microcarpa*
87	6,10-dihydroxylchelerythrine	$C_{21}H_{20}NO_6^+$	*M. microcarpa*
88	6,10,12-trihydroxychelerythrine	$C_{21}H_{20}NO_7^+$	*M. microcarpa*
89	6,10,12-trihydroxy-3-demethyldihydro-benzophenanthridinium	$C_{21}H_{22}NO_7^+$	*M. microcarpa*
90	6,10-dihydroxyl-3-demethyldihydro-benzophenanthridium	$C_{21}H_{22}NO_6$+	*M. microcarpa*
91	(±)-macleayins A	$C_{41}H_{36}N_2O_9$	*M. cordata*
92	(±)-macleayins B	$C_{40}H_{32}N_2O_9$	*M. cordata*
93	(±)-macleayins C	$C_{32}H_{31}NO_9$	*M. cordata*
94	(±)-macleayins D	$C_{23}H_{22}N_2O_5$	*M. cordata*
95	(±)-macleayins E	$C_{22}H_{18}N_2O_5$	*M. cordata*
96	6-acetonylnordihydrochelerythrine	$C_{23}H_{21}NO_5$	*M. cordata*
97	6-hydroxymethyldihydrosanguinarine	$C_{21}H_{17}NO_5$	*M. cordata*
98	7-demethyldihydrochelerythrine	$C_{20}H_{17}NO_4$	*M. cordata*
99	6-hydroxyethyldihydrochelerythrine	$C_{23}H_{24}NO_5^+$	*M. cordata*
100	cheilanthifoline	$C_{19}H_{19}NO_4$	*M. cordata*
			M. microcarpa
101	scoulerine	$C_{19}H_{21}NO_4$	*M. cordata*
102	tetrahydropalmatine	$C_{21}H_{25}NO_4$	*M. cordata*
103	13-hydroxyl-9-glu-scoulerine	$C_{25}H_{32}NO_{10}$	*M. cordata*
104	3-glu-scoulerine	$C_{25}H_{30}NO_9$	*M. cordata*
105	tetrahydroberberrubine/nandinine	$C_{19}H_{19}NO_4$	*M. cordata*
106	tetrahydrocolumbamine	$C_{20}H_{23}NO_4$	*M. cordata*
107	stylopine	$C_{19}H_{17}NO_4$	*M. cordata*
108	tetrahydroberberine	$C_{20}H_{21}NO_4$	*M. cordata*
109	dehydrocheilanthifoline	$C_{19}H_{16}NO_4^+$	*M. cordata*
110	coptisine	$C_{19}H_{14}NO_4^+$	*M. cordata*
			M. microcarpa
111	berberine	$C_{20}H_{18}NO_4^+$	*M. cordata*
			M. microcarpa
112	corysamine	$C_{20}H_{16}NO_4^+$	*M. cordata*
113	berberrubine	$C_{19}H_{15}NO_4^+$	*M. cordata*
			M. microcarpa
114	dehydrocorytenchine	$C_{20}H_{20}NO_4^+$	*M. cordata*
115	tetrahydropalmatrubine	$C_{20}H_{23}NO_4$	*M. cordata*
116	corytenchine	$C_{20}H_{23}NO_4$	*M. cordata*
117	jatrorrhizine	$C_{20}H_{20}NO_4^+$	*M. cordata*
118	columbamine	$C_{20}H_{20}NO_4^+$	*M. cordata*
119	13-hydroxylberberrabine	$C_{20}H_{20}NO_4^+$	*M. microcarpa*
120	13-hydroxylcoptisine	$C_{19}H_{14}NO_5^+$	*M. microcarpa*
121	13-methylberberine	$C_{21}H_{20}NO_4^+$	*M. microcarpa*
			M. cordata
122	13-hydroxylberberine	$C_{20}H_{18}NO_5^+$	*M. microcarpa*
123	berberrubine	$C_{19}H_{16}NO_4^+$	*M. microcarpa*
124	palmatine/berbericinine	$C_{21}H_{22}NO_4$	*M. microcarpa*
125	13-methoxyberberine	$C_{21}H_{20}NO_5^+$	*M. cordata*

126	thalifendine	$C_{19}H_{15}NO_4^+$	*M. cordata*
127	*N*-methyltetrahydrocoptisine	$C_{20}H_{20}NO_4^+$	*M. cordata*
128	*N*-methyltetrahydroberberine	$C_{21}H_{23}NO_4^+$	*M. cordata*
129	*N*-methyltetrahydroepiberberine	$C_{21}H_{24}NO_4^+$	*M. cordata*
130	*N*-methyltetrahydrocolumbamine	$C_{21}H_{26}NO_4^+$	*M. cordata*
131	*N*-methylstepholidine	$C_{20}H_{24}NO_4^+$	*M. cordata*
132	*N*-methylscoulerine/cyclanoline	$C_{20}H_{24}NO_4^+$	*M. cordata*
133	*N*-methyltetrahydropalmatine	$C_{22}H_{28}NO_4^+$	*M. microcarpa*
134	13-hydroxyl-*N*-methylcanadine	$C_{22}H_{26}NO_4^+$	*M. microcarpa*
135	13,14-dehydrogen-*N*-methylcoptsine	$C_{20}H_{18}NO_4$	*M. cordata*
136	*N*-methylcanadine	$C_{21}H_{24}NO_4^+$	*M. cordata*
137	14-hydroxyl-*N*-methylcoptisine	$C_{20}H_{20}NO_5$	*M. cordata*
138	14-hydroxyl-*N*-methylcanadine	$C_{21}H_{24}NO_5$	*M. cordata*
139	phellodendrine	$C_{20}H_{24}NO_4^+$	*M. cordata*
140	*N*-methylcheilanthifoline	$C_{20}H_{22}NO_4^+$	*M. cordata*
141	*N*-methylstylopine	$C_{20}H_{20}NO_4^+$	*M. cordata*
142	arnottianamide	$C_{21}H_{19}NO_6$	*M. microcarpa*
143	10-demethylmagoflorine	$C_{19}H_{22}NO_4^+$	*M. microcarpa*
144	2,3-methylenedioxy-7,10-dimethyl-7,8,9,10-tetrahydro-benzoquinoline	$C_{16}H_{17}NO_2$	*M. cordata*
145	2,3-methylenedioxy-7,10-dimethyl-8-carboxyl-benzoquinoline	$C_{17}H_{14}NO_4$	*M. cordata*
146	cavidilinine	$C_{19}H_{13}NO_4$	*M. cordata*
147	macleayine	$C_{19}H_{11}NO_8$	*M. cordata*

Table S2. Protoberberine-type alkaloids skeleton, exact theoretical masses ($[M+H]^+$), substituent groups(88 theoretical accurate *m*/*z* values).

skeleton	$[M+H]^+$	OCH_2O	OCH_3	OH	Glc
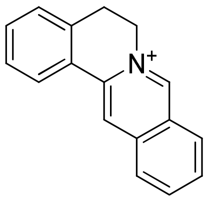	320.0917	2	0	0	0
	336.1230	1	2	0	0
	322.1074	1	1	1	0
	484.1602	1	1	0	1
	308.0917	1	0	2	0
	632.1974	1	0	0	0
	470.1446	1	0	1	1
	352.1543	0	4	0	0
	338.1387	1	3	1	0
	500.1915	0	3	0	1
	324.1230	0	2	2	0
	648.2287	0	2	0	2
	486.1759	0	2	1	1
	310.1074	0	1	3	0
	472.1602	0	1	2	0
	634.2130	0	1	1	2
	796.2659	0	1	0	3
	296.0917	0	0	4	0
	458.1446	0	0	3	1
	620.1974	0	0	2	2
	782.2502	0	0	1	3
	944.3030	0	0	0	4
	334.1074	2	0	0	0
	350.1387	1	2	0	0
	336.1231	1	1	1	0
	498.1759	1	1	0	1
	322.1074	1	0	2	0
	646.2131	1	0	0	2
	484.1603	0	1	0	1

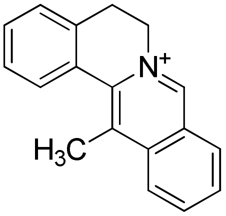	366.1700	0	4	0	0
	352.1544	0	3	1	0
	514.2072	0	3	0	1
	338.1387	0	0	2	2
	662.2444	0	2	0	2
	500.1916	0	2	1	1
	324.1231	0	1	3	0
	486.1759	0	1	2	1
	648.2287	0	1	1	2
	810.2816	0	1	0	3
	310.1074	0	0	4	0
	472.1603	0	0	3	1
	634.2131	0	0	2	2
	796.2659	0	0	1	3
	958.3187	0	0	0	4
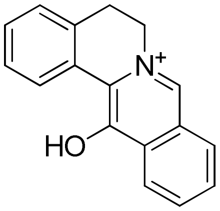	336.0866	2	0	0	0
	352.1179	1	2	0	0
	338.1023	1	1	1	0
	500.1551	1	1	0	1
	324.0866	1	0	2	0
	648.1923	1	0	0	2
	486.1395	1	0	1	1
	368.1492	0	4	0	0
	354.1336	0	3	1	0
	516.1864	0	3	0	1
	340.1179	0	2	2	1
	664.2236	0	2	0	2
	502.1708	0	2	1	1
	326.1023	0	1	3	0
	488.1551	0	1	2	1
	650.2079	0	1	1	2
	812.2608	0	1	0	3
	312.0866	0	0	4	0
	474.1395	0	0	3	1
	636.1923	0	0	2	2
	798.2451	0	0	1	3
	960.2979	0	0	0	4
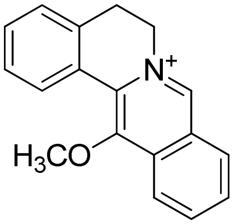	350.1023	2	0	0	0
	366.1336	1	2	0	0
	352.1180	1	1	1	0
	514.1708	1	1	0	1
	338.1023	1	0	2	0
	662.2080	1	0	0	2
	500.1552	1	0	1	1
	382.1649	0	4	0	0
	368.1493	0	3	1	0
	530.2021	0	3	0	1
	354.1336	0	2	2	0
	678.2393	0	2	0	2
	516.1865	0	2	1	1
	340.1180	0	1	3	0
	502.1708	0	1	2	1
	664.2236	0	1	1	2
	826.2765	0	1	0	3
	326.1023	0	0	4	0
	488.1552	0	0	3	1
	650.2080	0	0	2	2

	812.2608	0	0	1	3
	974.3136	0	0	0	4

Figure S1. MS/MS spectra of compound **4**, **5**, **6** and corresponding fragmentation behaviors.

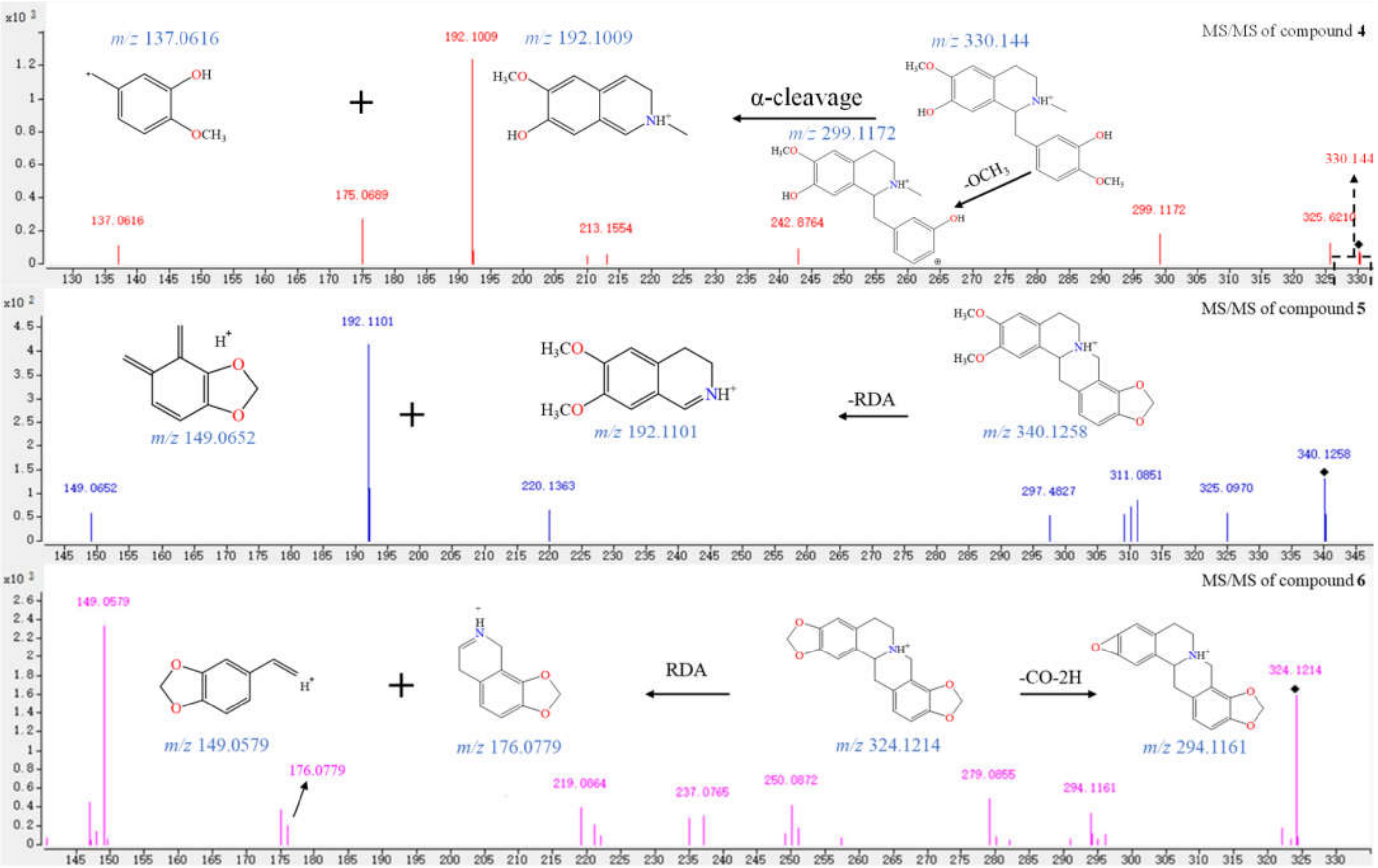

Figure S2. MS/MS spectra of compound **17** and **7** and corresponding fragmentation behaviors.

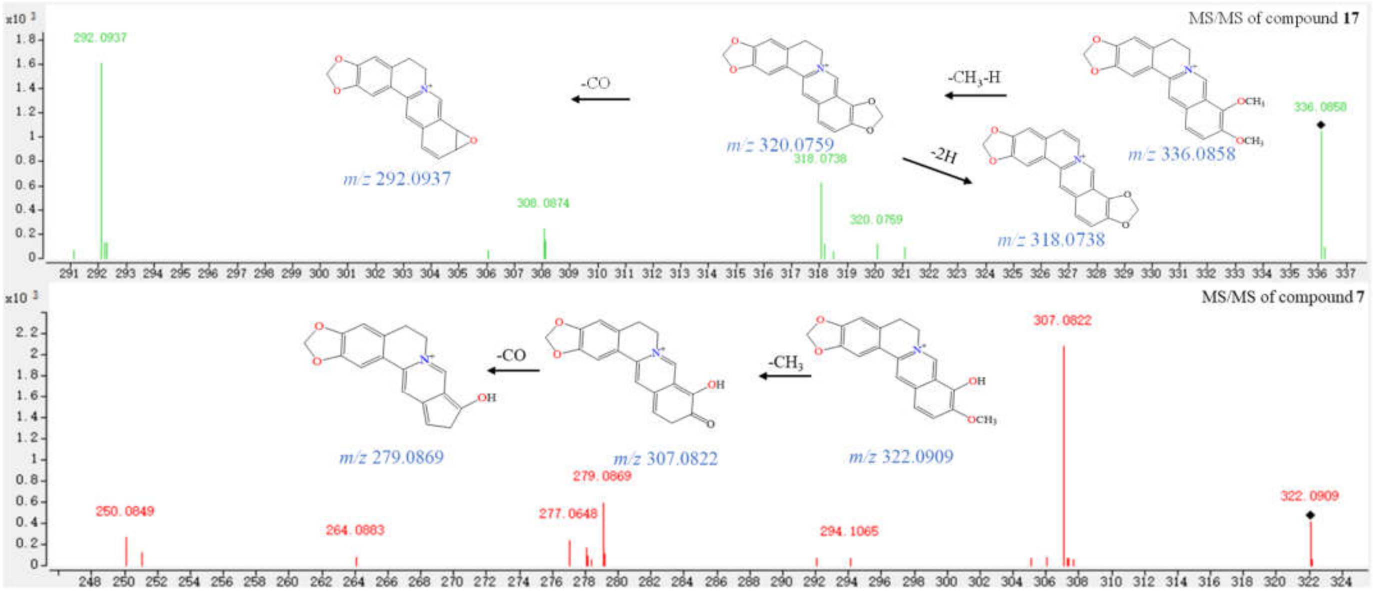

Figure S3. MS/MS spectra of compound **29** and **39** and corresponding fragmentation behaviors.

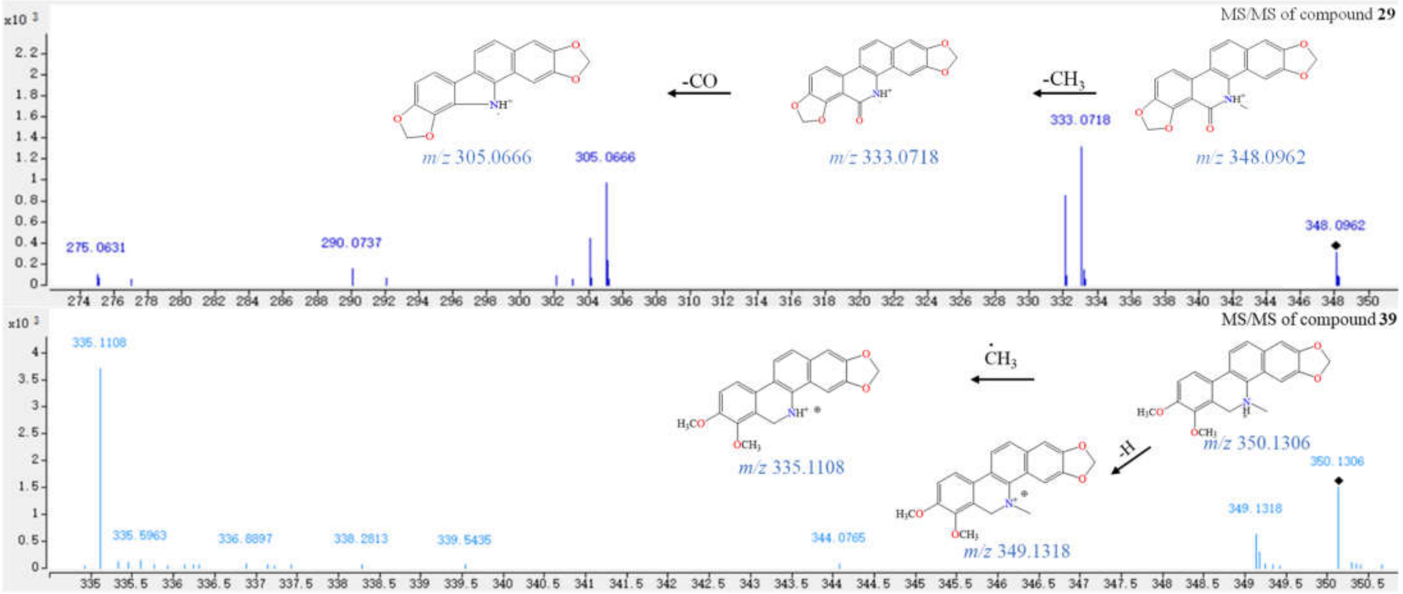

Figure S4. MS/MS spectra of the rest compounds.

1. *N. N*-dimethyl isouridine

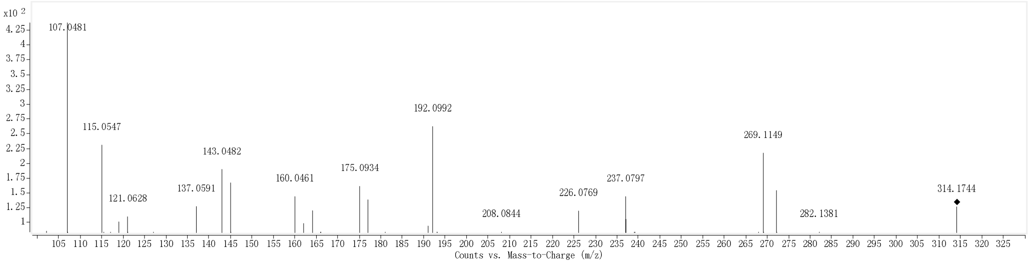

9. Protopine

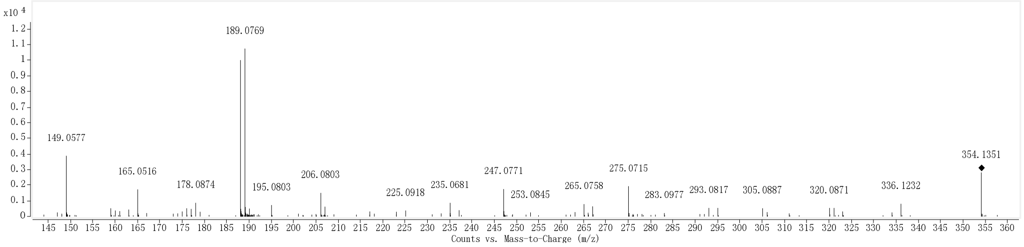

10. 13- hydroxyl- coptisine

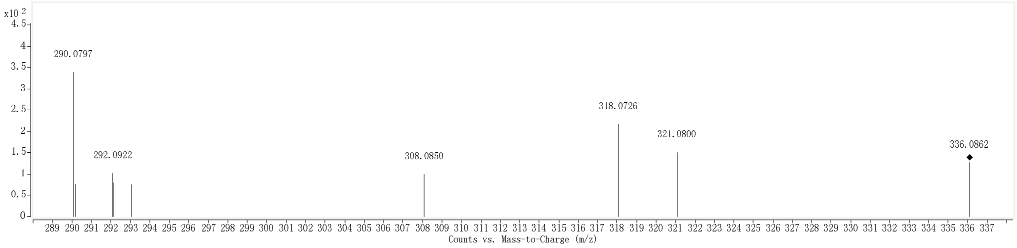

11. Allocryprotopine

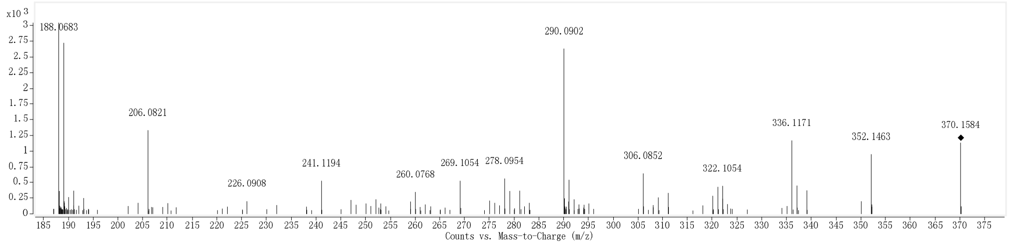

12. N-methylpyrophylline

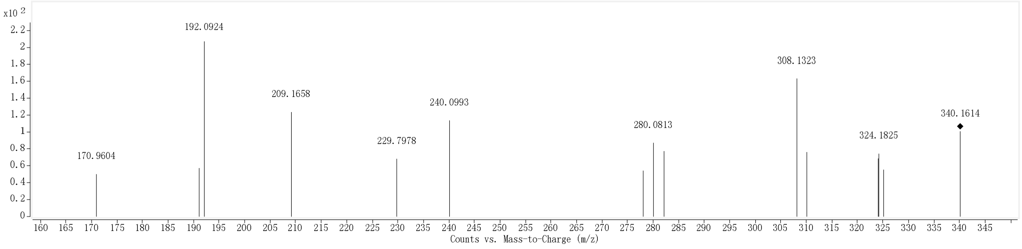

13. *N*-methyltetrahydropalmatine

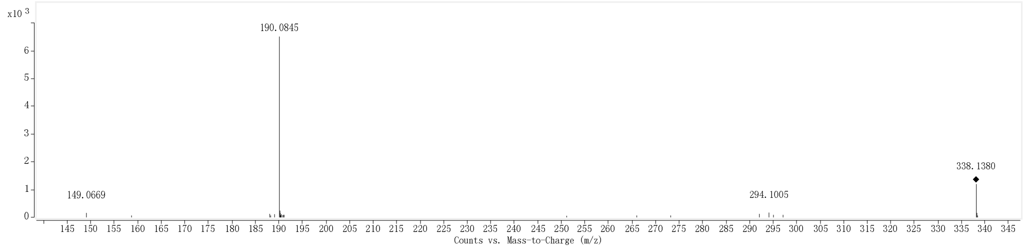

14. Didemethyl chelerythrine

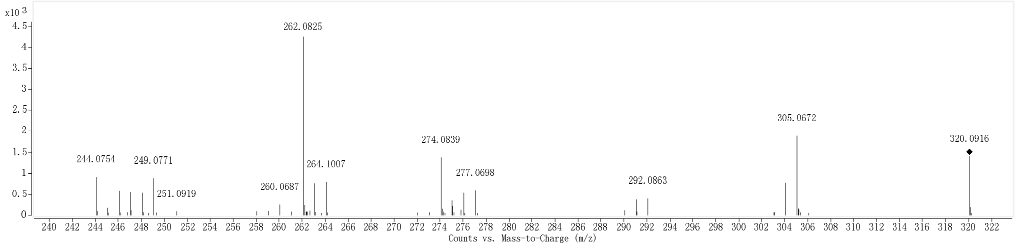

15. 7,8-dihydroberberine

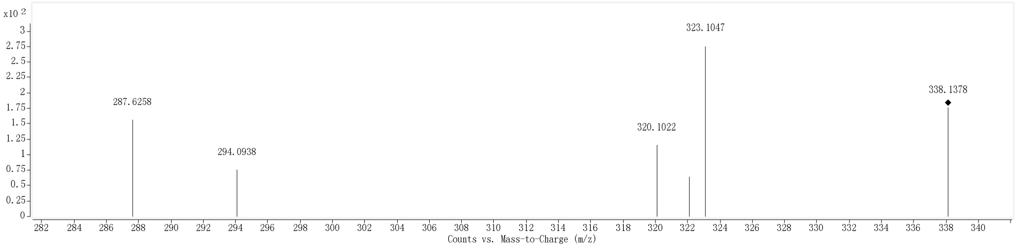

16. Demethylated chelerythrine

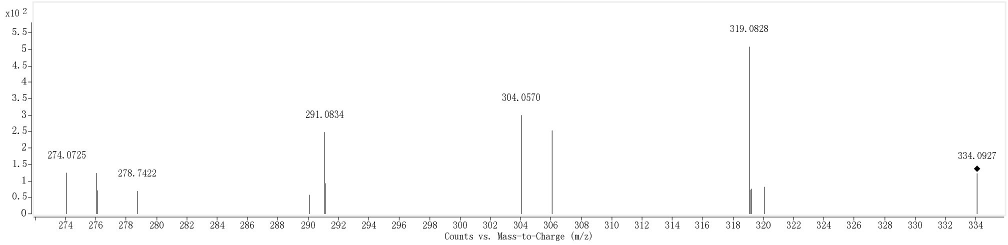

18. Dihydrosanguinarine

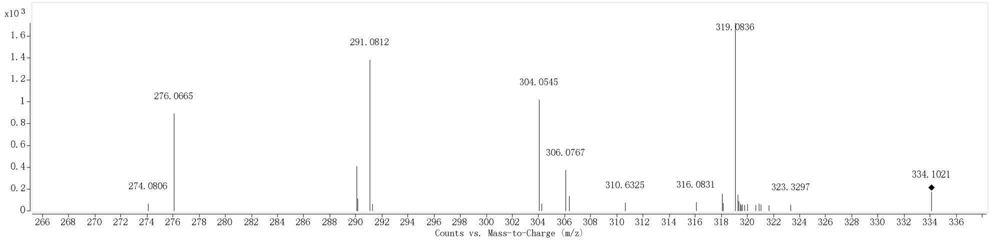

19. Isodimethylchelerythrine

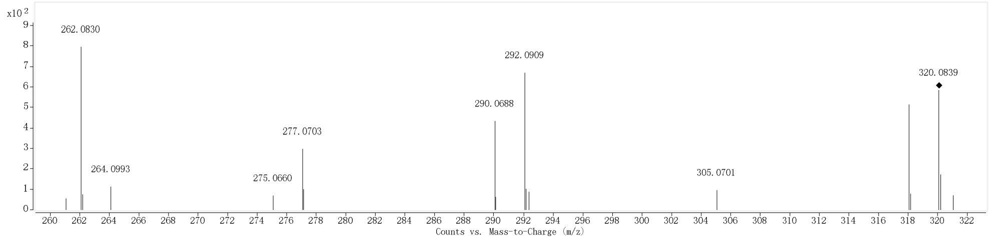

20. Isoprotopine

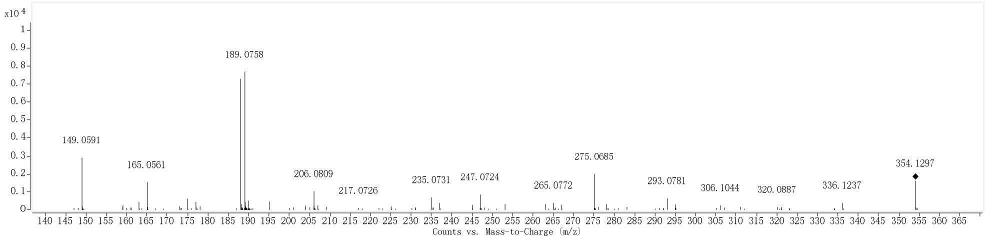

21. Sanguinarine

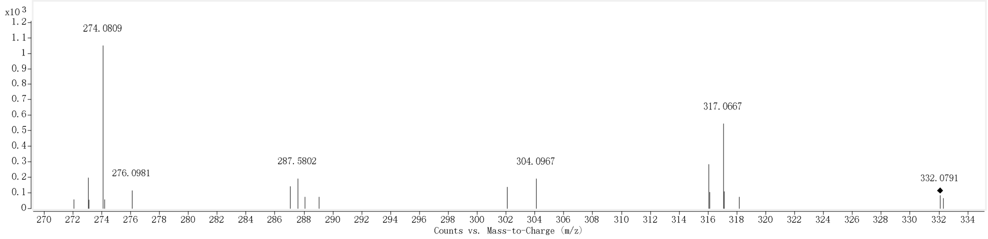

22. Oxychelerythrine

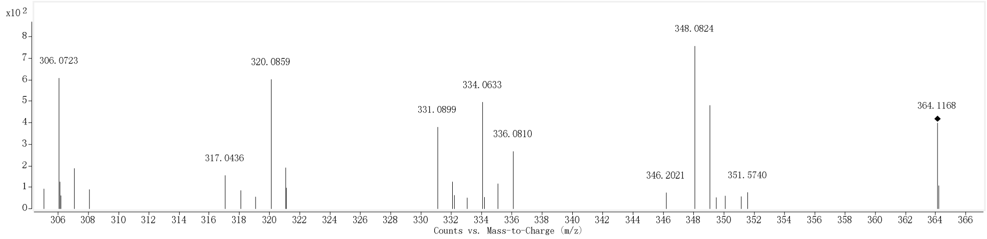

23. Demethyl-denitromethyl-benzophenanthridinium

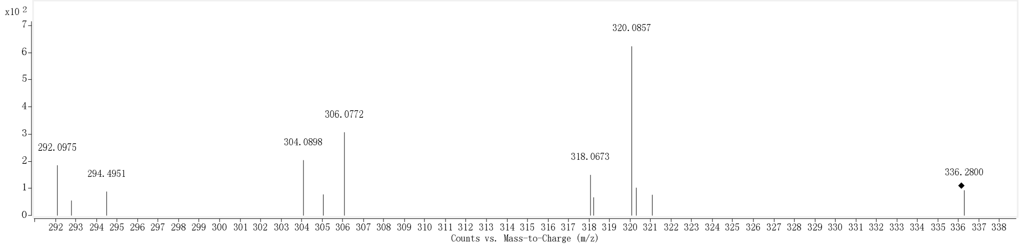

24. Demethyl-benzophenanthridinium

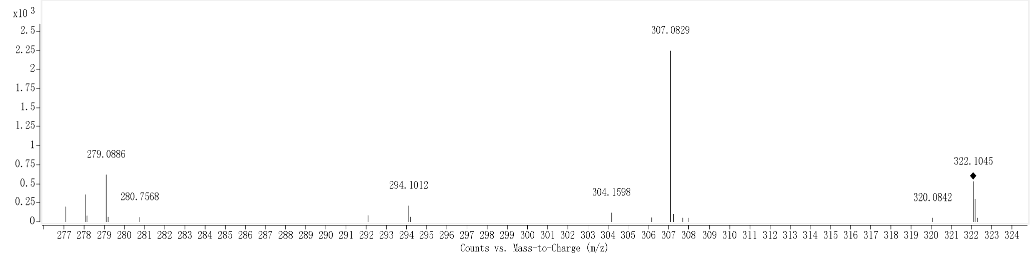

25. 6-acetoxy-dihydrosanguinarine

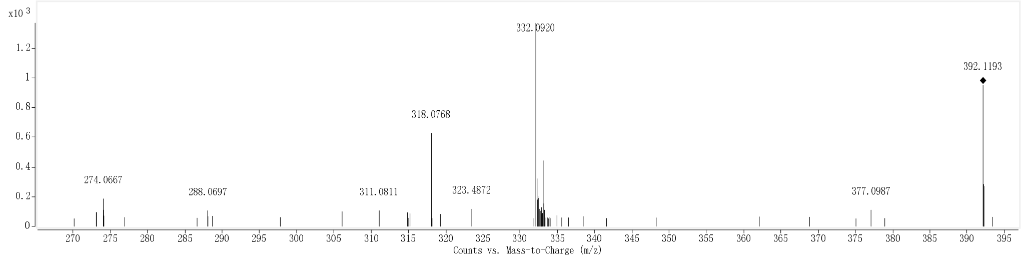

26. chelerythrine

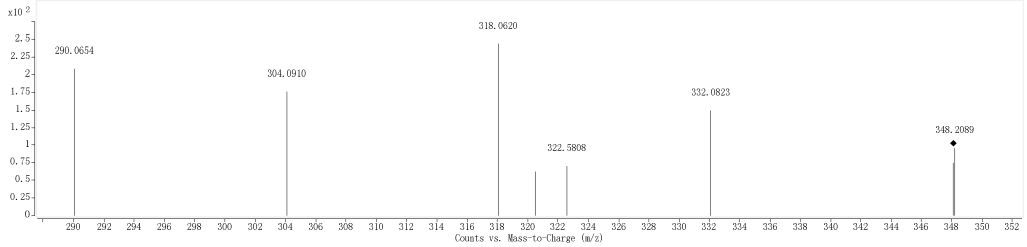

27. 6-acetoxy- dihydrochelervthrine

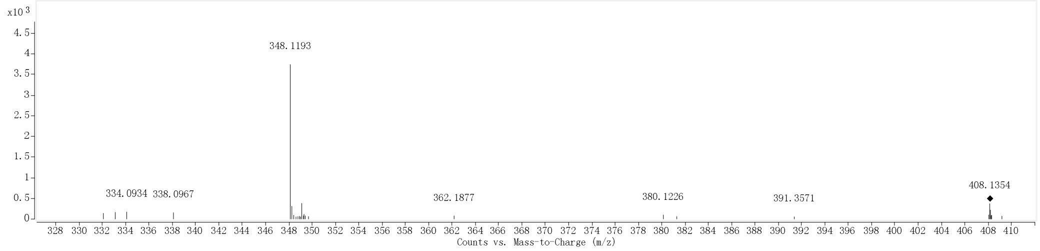

28. Maclekarpine E

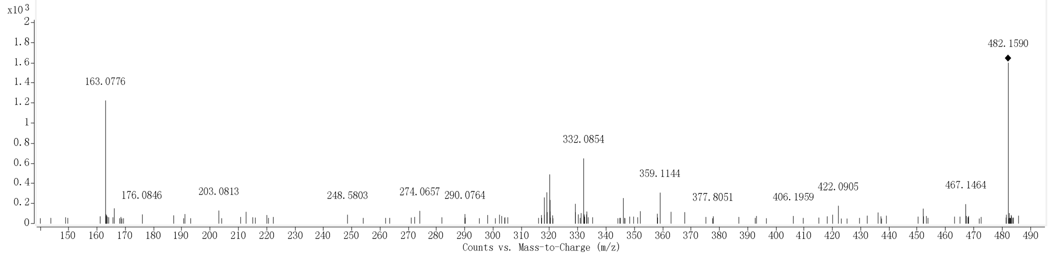

30. 6-amino-chelerythrine

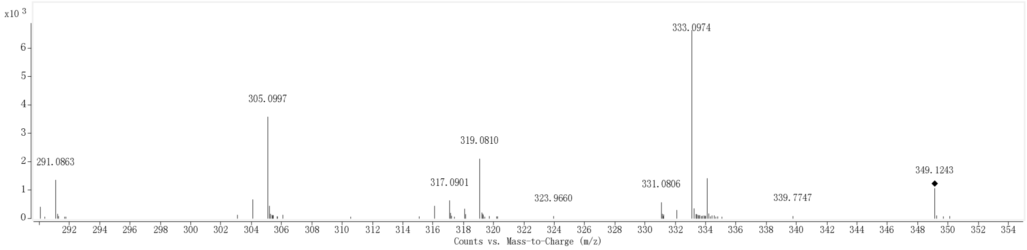

31. 6-methoxy- diazomethyl chelerythrine

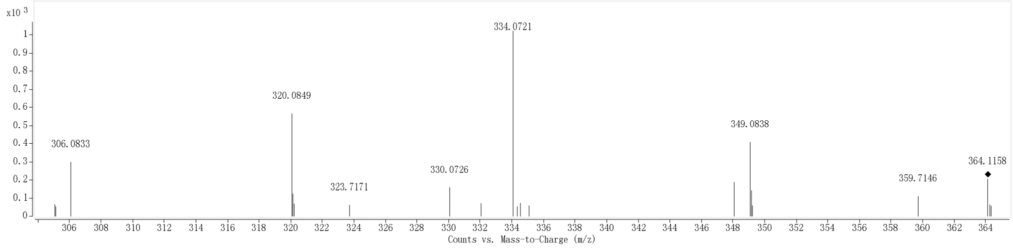

32. 10-methoxy-dihydrosanguinarine

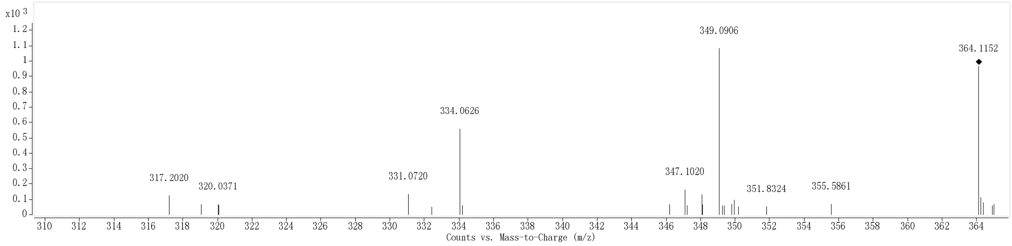

33. 10-methoxy-demethyl chelerythrine

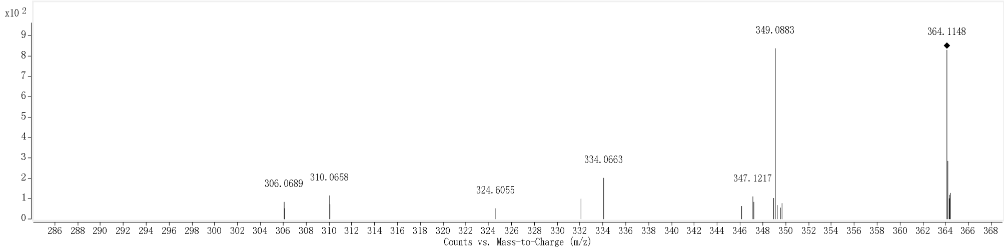

34. 6-methylol-dihydrosanguinarine

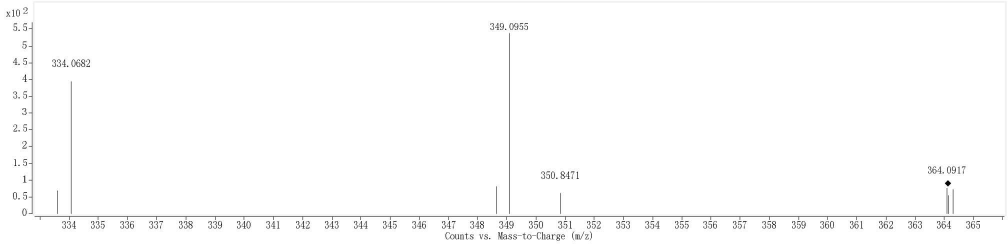

35. 6-ethoxy-dihydrosanguinarine

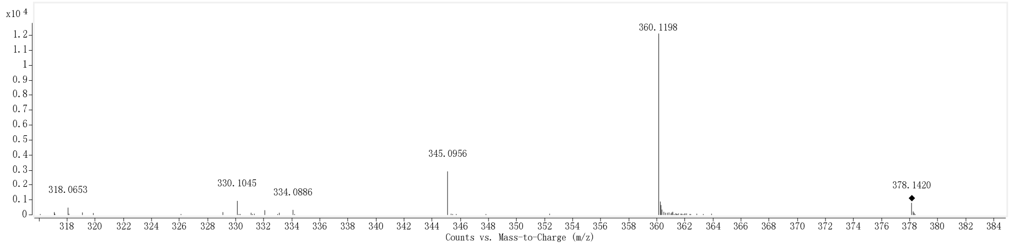

36. 6-hydroxyethylchelerythrine

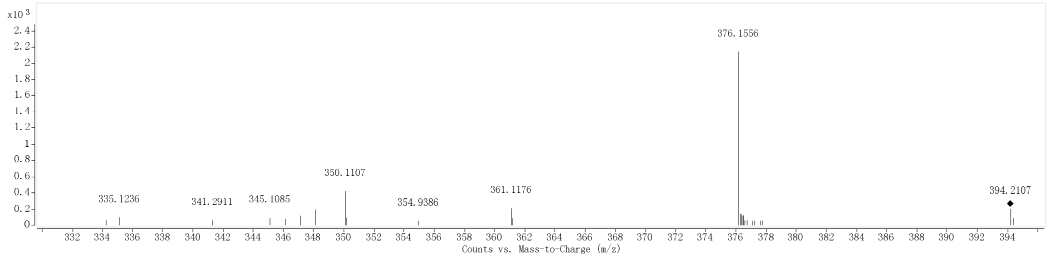

37. Diazomethylchelerythrine

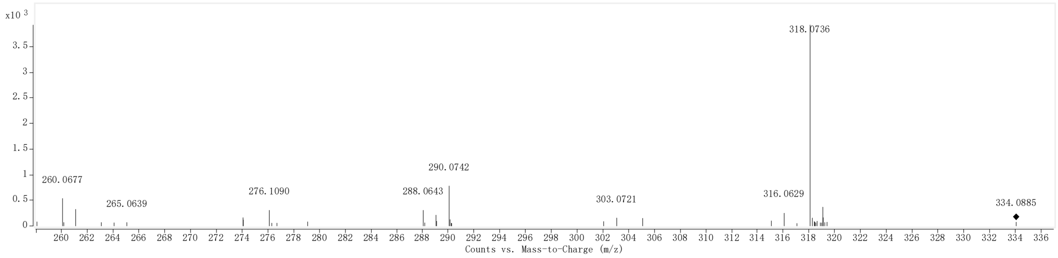

38. Diazomethylsanguinarine

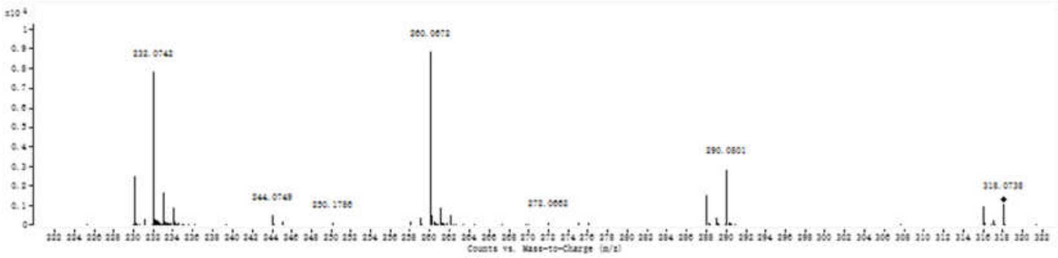

Supplement: Supplementary file 1 [file molecules-26-03851-s001.zip › molecules-1239359-supplementary.pdf]
